# Transcriptomic analysis of the host response to an iridovirus infection in Chinese giant salamander, *Andrias davidianus*

**DOI:** 10.1186/s13567-015-0279-8

**Published:** 2015-11-20

**Authors:** Yuding Fan, Ming Xian Chang, Jie Ma, Scott E. LaPatra, Yi Wei Hu, Lili Huang, Pin Nie, Lingbing Zeng

**Affiliations:** Yangtze River Fisheries Research Institute, Chinese Academy of Fishery Sciences, Wuhan, Hubei 430223 China; State Key Laboratory of Freshwater Ecology and Biotechnology, Institute of Hydrobiology, Chinese Academy of Sciences, Wuhan, Hubei 430072 China; Research Division, Clear Springs Foods, Inc., PO Box 712, Buhl, ID 83316 USA; Freshwater Aquaculture Collaborative Innovation Center of Hubei Province, Huazhong Agricultural University, Wuhan, Hubei 430223 China

## Abstract

The emergence of an infectious viral disease caused by the Chinese giant salamander iridovirus (GSIV) has led to substantial economic losses. However, no more molecular information is available for the understanding of the mechanisms associated with virus–host interaction. In this study, de novo sequencing was used to obtain abundant high-quality ESTs and investigate differentially-expressed genes in the spleen of Chinese giant salamanders that were either infected or mock infected with GSIV. Comparative expression analysis indicated that 293 genes were down-regulated and 220 genes were up-regulated. Further enrichment analysis showed that the most enriched pathway is “complement and coagulation cascades”, and significantly enriched diseases include “inherited thrombophilia”, “immune system diseases”, “primary immunodeficiency”, “complement regulatory protein defects”, and “disorders of nucleotide excision repair”. Additionally, 30 678 simple sequence repeats (SSRs) from all spleen samples, 26 355 single nucleotide polymorphisms (SNPs) from the spleens of uninfected animals and 36 070 SNPs from the spleens of infected animals were detected. The large amount of variation was specific for the Chinese giant salamanders that were infected with GSIV. The results reported herein provided significant and new EST information that could contribute greatly in investigations into the molecular functions of immune genes in the Chinese giant salamander.

**Electronic supplementary material:**

The online version of this article (doi:10.1186/s13567-015-0279-8) contains supplementary material, which is available to authorized users.

## Introduction

Amphibians are an important evolutionary bridge between aquatic and terrestrial vertebrates [1]. The Chinese giant salamander, *Andrias davidianus*, is the largest extant amphibian species and is considered a living fossil because it has existed for more than 350 million years [2]. The phylogenetic position of the Chinese giant salamander makes it an invaluable model for evolutionary and comparative studies. The Chinese giant salamander also has significant economic value as an edible delicacy and for medicinal purposes. However, in the past 50 years the Chinese giant salamander population has declined sharply due to deterioration of habitat, environmental pollution, climate change, infectious diseases and commercial trade [3, 4]. Currently, artificial breeding and culture is being used to protect the Chinese giant salamander. Approximately two million Chinese giant salamanders are bred annually in China.

With the rapid expansion of Chinese giant salamander farming, emerging infectious diseases have been increasing. Viral diseases, including the iridovirus, have caused major impacts to the Chinese giant salamander industry [5, 6]. The economic losses caused by the Chinese giant salamander iridovirus (GSIV) reached 300 million RMB (48 million USD) in 2010. There is an urgent need to understand the immune system of the Chinese giant salamander and the pathogenic mechanism(s) associated with a GSIV infection. In previous studies, the morphogenesis, pathological changes, rapid detection methods and virion-associated viral proteins of GSIV have been reported [7–12]. Although a ranavirus-induced thymus cDNA library and two immune organs (skin and spleen) from the healthy Chinese giant salamanders were sequenced [13, 14], and several immune genes were reported [15–17], the molecular information available is still limited for the Chinese giant salamander and that has hindered the understanding of the molecular mechanisms associated with viral infection and virus–host interactions.

Next-generation sequencing technology, such as de novo transcriptome sequencing, can be used for large-scale efficient and economical production of ESTs, and has become an important method for studying non-model species [18, 19]. Transcriptome sequencing facilitates functional genomic studies, including global gene expression, novel gene discovery, assembly of full-length genes, simple sequence repeats (SSRs) and single nucleotide polymorphism (SNP) discovery [20]. This technology has also been used widely in comparative transcriptomics to identify differences in transcript abundance among different developmental stages and under different treatment conditions [21, 22]. In the present study, the spleen transcriptome of the Chinese giant salamander was sequenced by de novo sequencing technology, and a comparative analysis of transcriptome data was performed between a control and a group infected with GSIV. The results provided a significant amount of information on the genes in the Chinese giant salamander, and suggested the conservation and divergence of several important immune signaling pathways. The different genes expressed and enrichment analysis of pathways could contribute significant new information regarding the pathogenic mechanism(s) of the virus and the interaction(s) of the virus and the host.

## Materials and methods

### Chinese giant salamanders, viral challenge and sample collection

Chinese giant salamanders (average weight, ~180 g) were obtained from the research farm of the Yangtze River Fisheries Research Institute in Wuhan, China. Prior screening indicated that these animals were free of GSIV. All the salamanders were kept in aerated, tap water supplied tanks at 20 °C and fed with diced bighead carp (*Hypophthalmichthys nobilis*) for 2 weeks prior to the experiment. The virus suspension used to infect a portion of the salamanders was obtained from GSIV-infected EPC cells. The fish in infected group (GS_TS) were injected intramuscularly with 0.2 mL of the GSIV suspension (5 × 10^7^ TCID50/mL), and fish in control group (GS_CS) were injected intramuscularly with equal volume of DPBS (Sigma). The spleens from three individual salamanders from each group were collected at 48 h post-injection.

### RNA isolation and cDNA synthesis

Total RNA was extracted from the spleens using TRIzol^®^ Reagent (Invitrogen, USA). Samples of the three individuals from each treatment group were pooled in equal amounts to generate one RNA sample per group. These two RNA samples were sent to Shanghai Majorbio Bio-pharm Biotechnology Co., Ltd. (Shanghai, China) for the cDNA library construction and Illumina deep sequencing.

### cDNA library construction and Illumina deep sequencing

Two cDNA libraries were prepared using the TruseqTM RNA sample prep Kit (Illumina, San Diego, CA, USA) following the manufacturer’s instructions. Briefly, poly (A)+ RNA was purified from 5 μg of pooled total RNA using oligo (dT) magnetic beads, sheared into short fragments, and primed for cDNA library synthesis using the TruSeq RNA sample preparation kit according to the manufacturer’s instructions (Illumina). After quantitation using a TBS-380 minifluorometer (PicoGreen), the samples were clustered (TruSeq paired-end cluster kit v3-cBot-HS; Illumina) and sequenced on the HiSeq 2000 platform (100 bp, TruSeq SBS kit v3-HS 200 cycles; Illumina).

### Data analysis

The raw reads from the images were generated using Solexa GA pipeline 1.6. After the removal of low-quality reads, processed reads with an identity value of 95% and a coverage length of 100 bp were assembled using the Trinity de novo assembler [23]. The isogenes generated were compared with the NCBI non-redundant (nr) database using the BLASTx algorithm, with a cut-off E value of ≤10^−5^. GO terms were extracted from the best hits obtained from the BLASTx against the nr database using Blast2GO [24]. These results were then sorted by GO categories using in-house Perl scripts. BLASTx was also used to align unique sequences to the Swiss-Prot database, Kyoto Encyclopedia of Genes and Genomes (KEGG) and Clusters of Orthologous Groups (COG) (with the e value of 10^−6^) to predict possible functional classifications and molecular pathways [25].

### Differential expression analysis

To identify differentially expressed genes/isogenes between infected and uninfected groups, genes/isogenes expression levels were measured by using numbers of fragments per kilobase of transcript per million fragments sequenced (FPKM) [26], similar to RPKM (reads per kilobase of gene model exon per million mapped reads) measure used earlier [27]. The differential expression analysis was carried out using RSEM [28] and edgeR [29] softwares. For each gene/isogene, the *p* value was computed, and then Benjamini–Hochberg false discovery rate (FDR) was applied to correct the results for *p* value. The transcripts that were increased or decreased at an estimated absolute log_2_-fold change of >1 and FDR adjusted *p* value ≤ 0.05 were considered to be differentially expressed.

### Identification of EST-SSR motifs and EST-SNPs

MSATCOMMANDER V. 0.8.2 [30] was used to analyze the microsatellite (SSR) distribution. The minimum number of repeats for SSR detection was six for di-SSRs and four repeats for tri-, tetra-, penta-, and hexa-SSRs. The open reading frame (ORF) and untranslated region (UTR) within the isotig were identified using Trinity [23]. The location of SSRs was estimated based on ORFs and UTRs. SSR-containing isotigs were annotated based on BLAST similarity searches. SNPs were detected based on alignment using BWA V. 0.5.9 [31] and SAMtools V. 0.1.18 [32]. From the “pileup” output of SAMtools, VarScan V.2.2.7 filtered SNPs based on the following criteria including (1) the total coverage and the number of reads to cover a candidate SNP (>8 reads); (2) the base quality where base calls with low Phred quality (<25) were removed from the coverage; and (3) frequency of mutated bases higher than 30% among all reads covering the position.

### Quantitative real-time PCR

Quantitative real time PCR was performed using iQ™ SYBR Green Supermix (Bio-Rad, Singapore) on a BIO-RAD CFX96 Real-Time System under the following conditions: 3 min at 95 °C, followed by 45 cycles of 15 s at 94 °C, 15 s at 55 °C and 30 s at 72 °C. Different genes including complement component C1R, C1S, C1S-like, C2, C3, C4, C5, C7, C8A and C9 were used for validation. An additional file shows the primer sequences used in this study (Additional file 1). The relative expression levels of the selected genes were normalized to β-Actin and calculated using 2^−ΔΔCt^ method.

## Results

### De novo sequencing and assembly

Two sequencing libraries were prepared from spleen samples obtained from control (GS_CS) and GSIV-infected (GS_TS) Chinese giant salamanders that were sequenced using an Illumina Hiseq 2000. In total, 122.48 million raw reads were generated from GS_CS and 154.75 million for GS_TS. The data was refined by discarding low-quality reads that contained unknown bases or whose length was lower than 20 nt after removal of the adaptors and low-quality bases. The resulting high-quality reads numbered 113.45 million and 143.78 million for the GS_CS and GS_TS samples, respectively. The total length of these reads was 9.6 × 10^9^ and 11.97 × 10^9^ bp for GS_CS and GS_TS samples, respectively and the Q20 percentage (the percentage of sequences with a sequencing error rate lower than 1%) was over 98% for both samples (Table 1). All high-quality reads were deposited in the National Central for Biotechnology Information (NCBI) and can be accessed under the accession number SRP047398.

**Table 1:** Summary of sequencing results.

	GSIV-infected	Control
Total raw reads	154 748 118	122 483 450
Total clean reads	143 776 686	113 450 692
Total clean nucleotides (bp)	11 967 607 097	9 600 577 224
Q20 percentage	98.44%	98.43%

**Figure 1:**
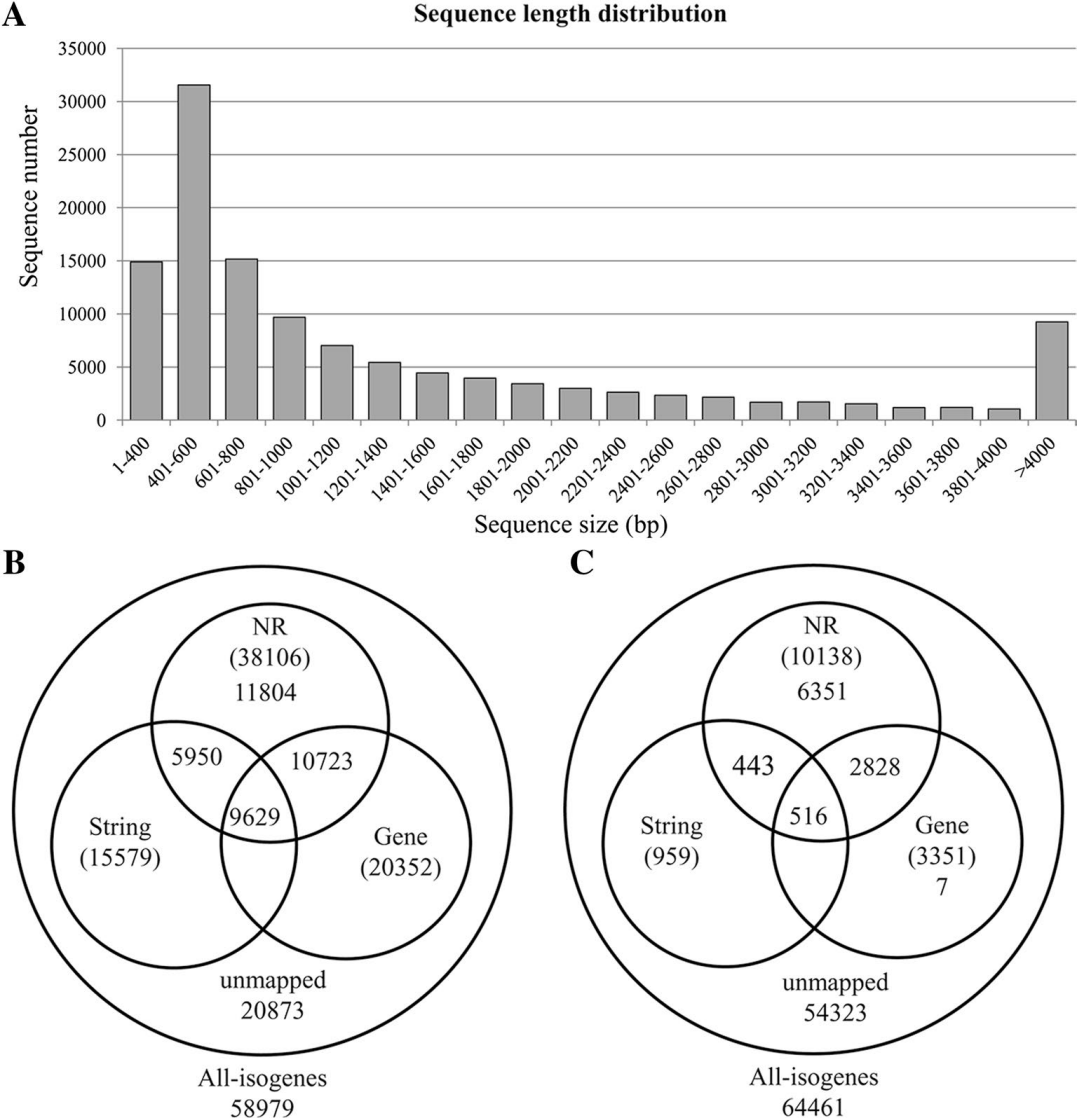
**Sequence length distribution and number of isogenes blasted to NR, String and Gene databases. A** The length distribution of the isogenes. **B** Number of protein sequences blasted to NR, String and Gene databases. **C** Number of DNA sequences without ORFs blasted to NR, String and Gene databases.

De novo assembly was performed using Trinity that resulted in 80 367 genes and 123 440 isogenes. The total length was 182 916 518 bp, with an average length of 1481 bp (Table 2). Each isogene was longer than 351 bp, and 71 295 (57.76%) of the isogenes were 350–1000 bp. Additionally, 27 826 (22.5%) of the isogenes were longer than 2000 bp. The size distribution of isogenes is shown in Figure 1A.

**Table 2:** Summary of assembly results.

Type	Number
Total gene	80 367
Total isogenes	123 440
Total residues	182 916 518
Average length	1481.83
Largest isogene	18 965
Smallest isogene	351

### Functional annotation and classification

All assembled high-quality isogenes had their ORFs predicted using Trinity. A total of 58 979 (47.8%) isogenes contained an ORF. The predicted protein sequences were blasted against NR (non-redundant protein sequences in NCBI), String and Gene databases using BLASTP with a cut-off E value of 10^−5^. There were 38 106 (64.6%) isogenes with homologous sequences in at least one of the databases. Among them, 38 106 (64.6%), 15 579 (26.4), and 20 352 (34.5%) isogenes were found in NR, String and Gene databases, respectively. A total of 9629 (16.3%) isogenes were found in all three databases, while 20 873 (35.4%) isogenes were not identified (Figure 1B).

The ORFs of 64 461 (52.2%) isogenes could not be predicted, and the DNA sequences of 64 461 isogenes were blasted against the NR, String and Gene databases using BLASTX with a cut-off E value of 10^−5^. There were 10 138 (15.7%) isogenes with homologous sequences in at least one of above databases. Among them, 10 138 (15.7%), 959 (1.5%), and 3351 (5.2%) isogenes were found in the NR, String and Gene databases, respectively. A total of 516 (0.8%) isogenes were found in all three databases, while 54 323 (84.3%) isogenes were not identified (Figure 1C).

**Figure 2:**
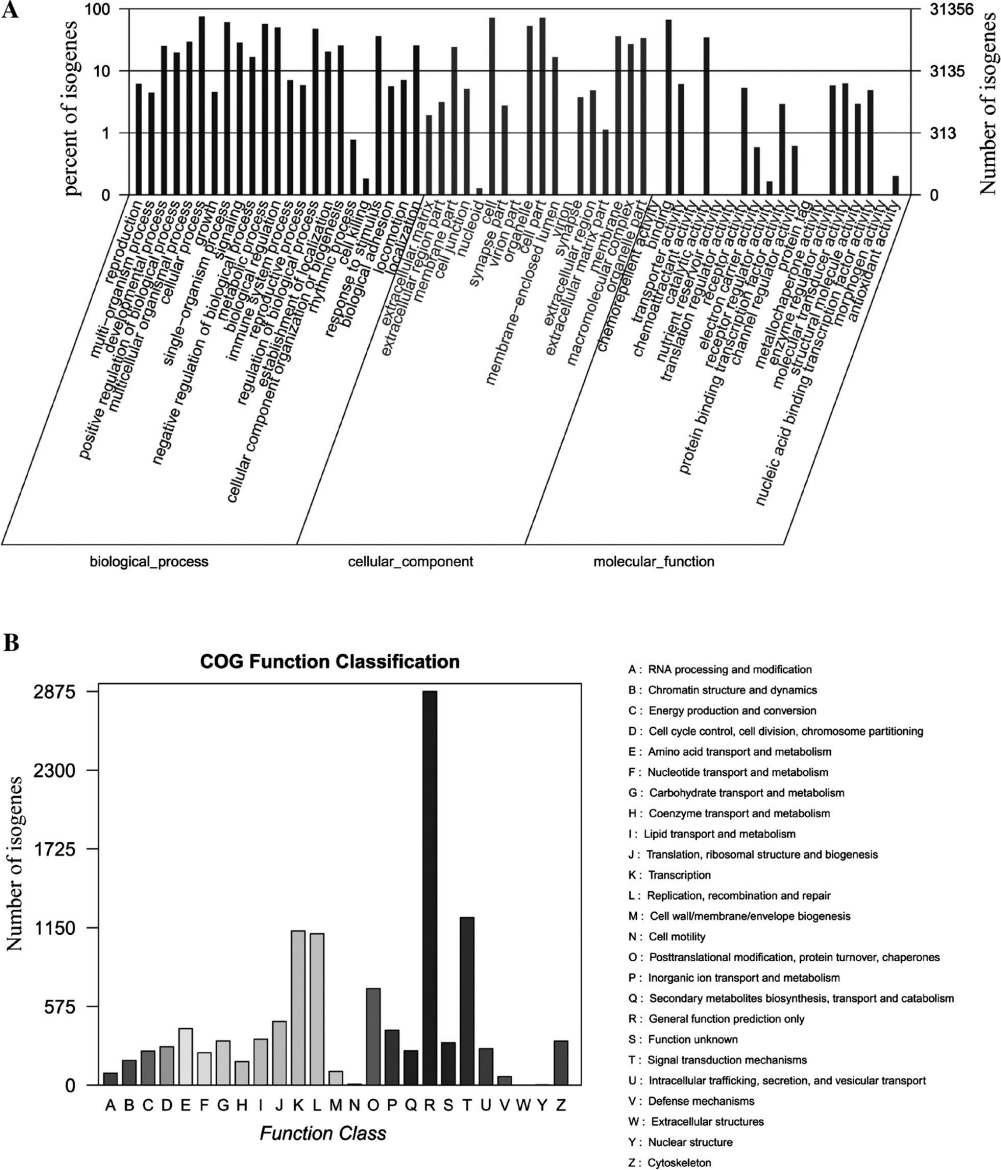
**GO functional annotation and COG function classification.** **A** Go functional annotation. Isogenes with the best BLAST hits were aligned to GO database. All 31 356 isogenes were assigned to at least one GO term and were grouped into three main GO categories and 61 sub-categories. Right y axis represents number of isogenes in a category. Left y axis indicates percentage of a specific category of isogenes in each main category. **B** COG classification of putative proteins. All 16 538 putative proteins showing significant homology to those in the COG database were functionally classified into 25 families. The y axis indicates number of isogenes in a specific functional cluster.

Based on NR annotations, the Gene Ontology (GO) classification system was used to classify the possible functions of the isogenes. A total of 31 356 (25.4%) isogenes were successfully assigned to at least one GO term annotation and were classified into three main categories including biological process, cellular component and molecular function (Figure 2A). For biological process, the top six largest categories were cellular process (23 645), single-organism process (19 125), metabolic process (17 849), biological regulation (15 665), regulation of biological process (14 954) and response to stimulus (11 342). For the cellular component category, the top three largest categories were cell (22 548), cell part (22 544) and organelle (16 663). Only a few isogenes belonged to the virion (29), and virion part (24) sub-categories. Interestingly, for the molecular function category, 20 914 and 10 902 isogenes were classified into the sub-categories “binding” and “catalytic activity”, respectively.

Isogenes sequences in our transcriptome library were also analyzed by the Clusters of Orthologous Groups of proteins (COG). Out of 38 106 NR hits, 7719 (20.3%) of the sequences showed a COG classification (Figure 2B). The largest category was “general function prediction only” (2875 of 7719 isogenes or 37.2%), followed by “signal transduction mechanisms” (1224 isogenes, 15.9%), “transcription” (1126 isogenes, 14.6%), “replication, recombination and repair” (1105 isogenes, 14.3%) and “post-translational modification, protein turnover, chaperones” (706 isogenes, 9.1%). The sub-categories “extracellular structures” (0, 0), “nuclear structure” (4, 0.05%) and “cell motility” (8, 0.1%) had the fewest related genes. Additionally, 310 (4.02%) isogenes were annotated as “function unknown”.

The 38 106 annotated sequences were mapped to the reference canonical pathways in the Kyoto Encyclopedia of Genes and Genomes (KEGG). Among those, 23 712 isogenes were assigned to 317 KEGG pathways (Additional file 2). Of the 23 712 isogenes, 2195 (9.26%) were related to metabolic pathways, 784 (3.31%) to pathways in cancer, 750 (3.16%) to PI3K-Akt signaling pathway, 643 (2.71%) to MAPK signaling pathway, 614 (2.59%) to neuroactive ligand-receptor interaction, and 604 (2.55%) to HTLV-I infection.

### Differential expression analysis

**Figure 3:**
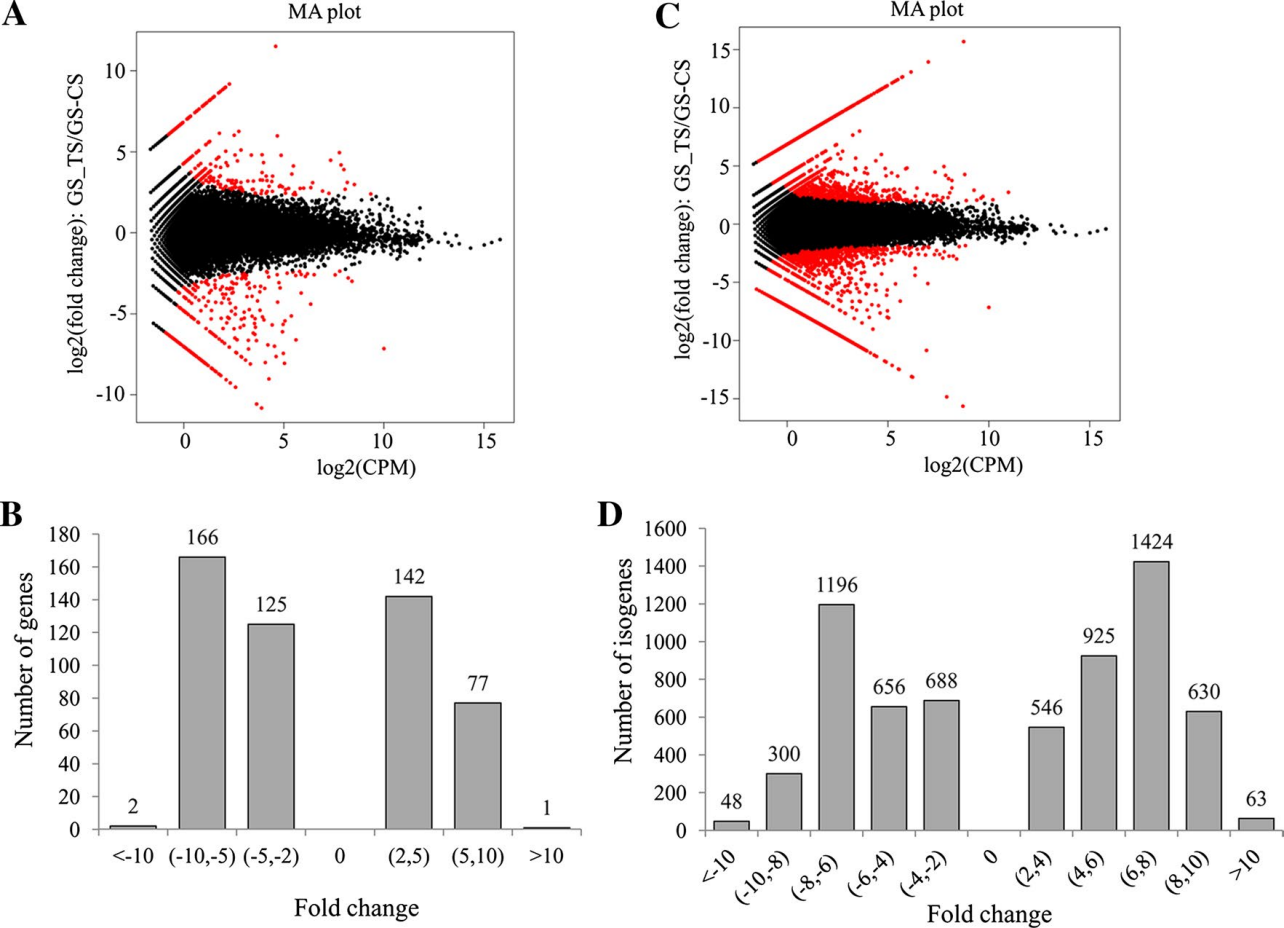
**Identification of differentially expressed genes/isogenes between infected and uninfected groups. A** The expression levels of differentially expressed genes. **B** The distribution of differentially expressed genes. **C** The expression levels of differentially expressed isogenes. **D** The distribution of differentially expressed isogenes. Differentially expressed genes or isogenes were determined using a threshold of FDR ≤ 0.001 and |log_2_Rario| > 1. Redspots represent differentially expressed genes or isogenes. Black spots represent genes or isogenes that didn’t show obvious changes in the GSIV-infected Chinese giant salamander.

To identify differential expression changes between GS_CS and GS_TS samples, FPKM method was used to calculate the expression levels of genes and isogenes. The results showed that 293 genes were down-regulated and 220 genes were up-regulated with an FDR < 0.05 and ratios larger than 2 (Figures 3A and B). Among them, 102 down-regulated genes were detected only in the GS_CS samples (Additional file 3A), and 67 up-regulated genes specific for the GS_TS samples (Additional file 3B). For the isogenes, 2888 down-regulated and 3588 up-regulated isogenes were identified (Figures 3C and D).

Enrichment analysis was conducted to help clarify the biological functions of all differentially expressed isogenes (DEGs) that were identified. All DEGs were mapped to each term of the GO database, and the GO terms with a corrected *P* value ≤0.05 were defined as significantly enriched in DEGs. The results indicated that 2493 DEGs were enriched in 433 GO terms (Additional file 4). Among these GO terms, “immune system process” (172 DEGs), “regulation of immune system process” (123 DEGs), “response to stimulus” (962 DEGs), “response to DNA damage stimulus” (78 DEGs), “lymphocyte activation” (45 DEGs), “T cell activation” (32 DEGs), “leukocyte migration” (30 DEGs) and “chemotaxis” (81 DEGs) were significantly enriched among DEGs compared with the whole transcriptome background.

**Figure 4:**
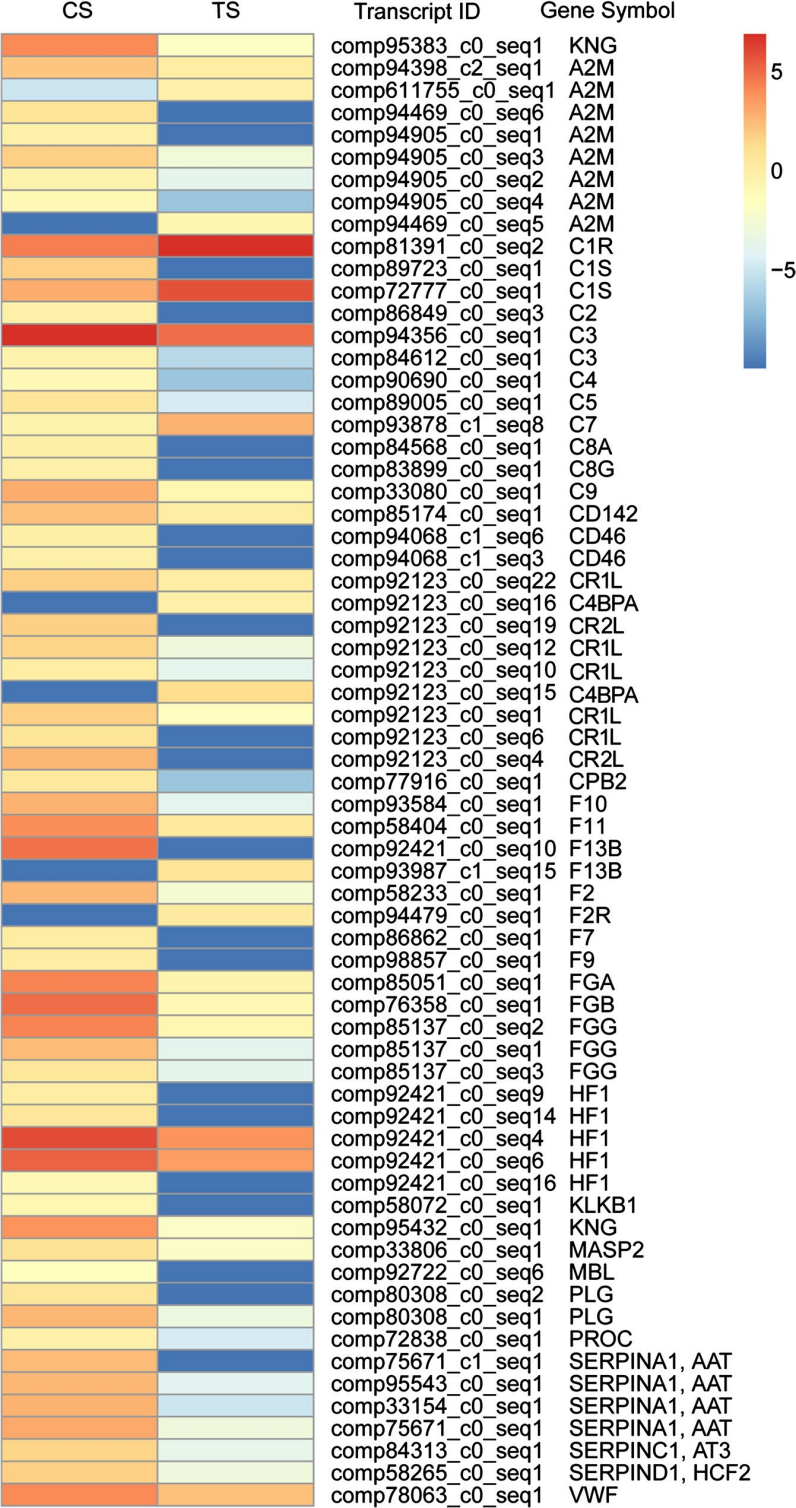
**The gene cluster for “complement and coagulation cascades” pathway.** 66 differentially expressed genes (DEGs) were enriched in the pathway, with 7 DEGs up-regulated and 59 DEGs down-regulated.

**Figure 5:**
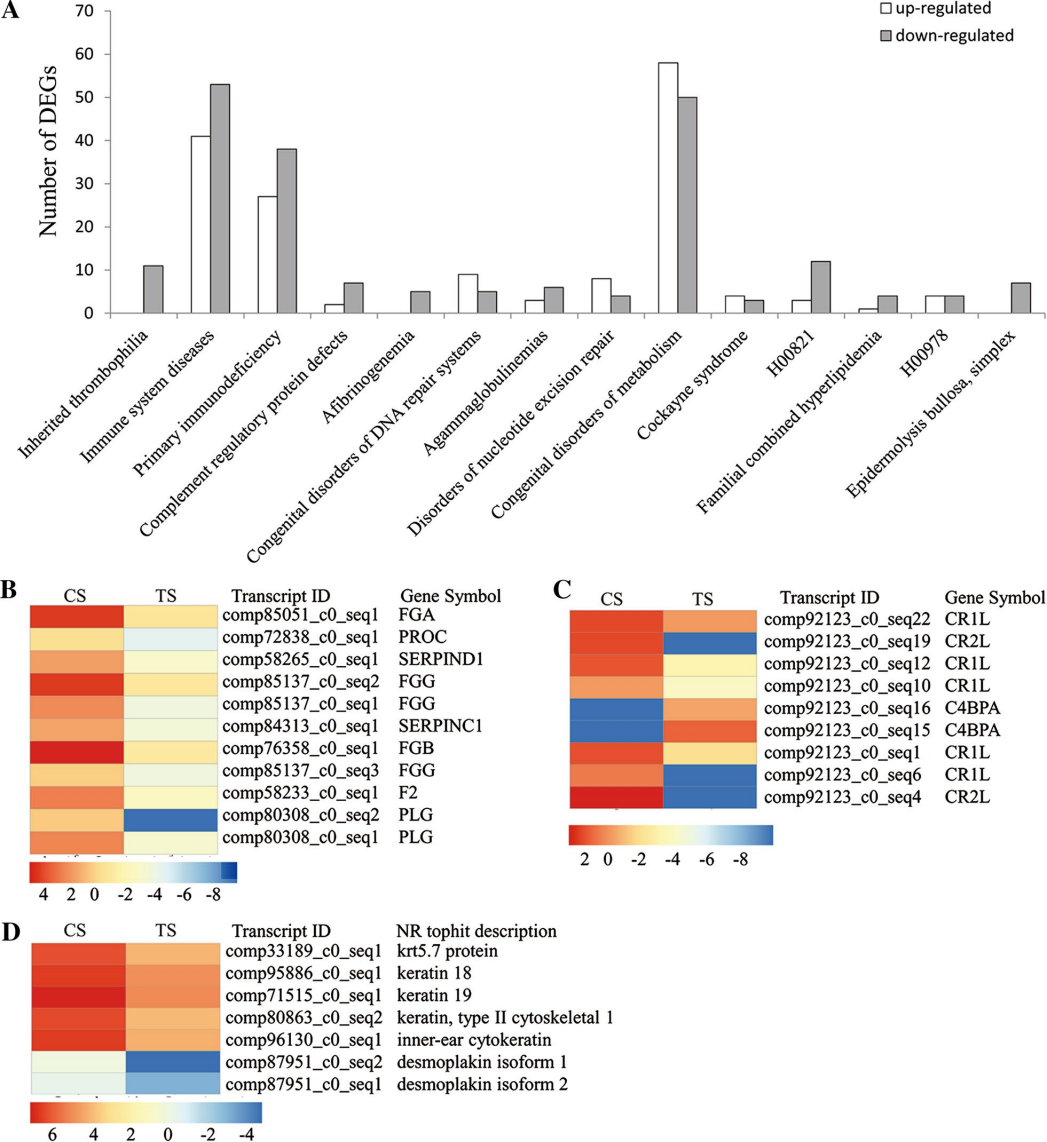
**Number and gene clusters of differentially expressed isogenes in KEGG diseases. A** KEGG enrichment analysis showed significantly enriched disease. The y axis indicates numbers of DEGs enriched in KEGG diseases. The x axis represents disease terms. The black bar indicates down-regulated DEGs and the white bar represents up-regulated DEGs. **B** The gene cluster for inherited thrombophilia. **C** The gene cluster for complement regulatory protein defects. **D** The gene cluster for Epidermolysisbullosa, simplex. Those DEGs associated with inherited thrombophilia, complement regulatory protein defects and Epidermolysisbullosa, simplex diseases appeared strongly suppressed in the spleen of GSIV-infected Chinese giant salamanders.

We also performed an enrichment analysis of the KEGG pathways and diseases. These DEGs were significantly enriched in 7 pathways including “complement and coagulation cascades”, “hematopoietic cell lineage”, “*Staphylococcus aureus* infection”, “tight junction”, “viral myocarditis”, “transcriptional misregulation in cancer” and “allograft rejection” (Additional file 5). The most enriched pathway was “complement and coagulation cascades” (66 DEGs), with 7 DEGs up-regulated and 59 DEGs down-regulated (Figure 4). The significantly enriched disease terms included inherited thrombophilia (11 DEGs), immune system diseases (94 DEGs), primary immunodeficiency (71 DEGs), complement regulatory protein defects (9 DEGs), afibrinogenemia (5 DEGs), congenital disorders of the DNA repair systems (15 DEGs), agammaglobulinemias (9 DEGs), disorders of nucleotide excision repair (13 DEGs), congenital disorders of metabolism (112 DEGs), cockayne syndrome (7 DEGs), H00821 (15 DEGs), familial combined hyperlipidemia (5 DEGs), H00978 (8 DEGs) and epidermolysisbullosa, simplex (7 DEGs) (Additional file 5). Among those DEGs involved in immune system diseases, 41 DEGs were up-regulated and 53 DEGs were down-regulated (Figure 5A). Those DEGs associated with inherited thrombophilia, complement regulatory protein defects, afibrinogenemia, H00821, epidermolysisbullosa, and simplex diseases were strongly suppressed in the spleen of GSIV-infected Chinese giant salamanders (Figure 5).

### SSR and SNP discovery

**Figure 6:**
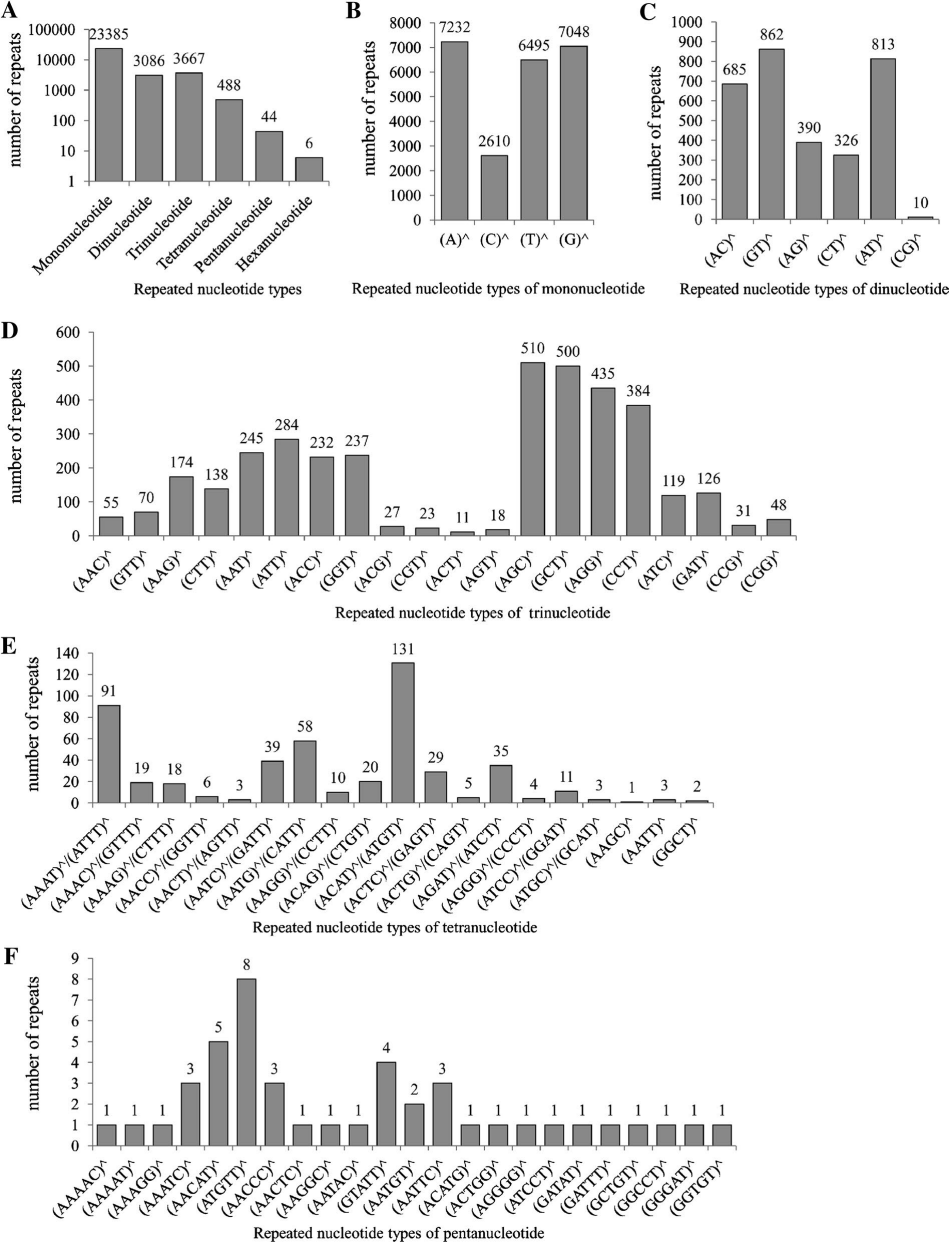
**Distribution of simple sequence repeats (SSR) among different nucleotide types in the spleen transcriptome.** **A** Distribution of repeated nucleotide types. **B** Distribution of repeated mononucleotide. **C** Distribution of repeated dinucleotide. **D** Distribution of repeated trinucleotide. **E** Distribution of repeated tetranucleotide. **F** Distribution of repeated pentanucleotide.

Simple sequence repeats (SSRs) have been shown to be an efficient tool for performing quantitative trait loci (QTL) analysis and constructing genetic linkage(s) due to their high diversity and abundance. In the present study, 30 678 SSRs were obtained from the transcriptomic information from the Chinese giant salamander. Among them, the most frequent repeat motifs were mononucleotide SSR motifs (76.2%), followed by trinucleotide (11.95%), dinucleotide (10.06%), tetranucleotide (1.6%), pentanucleotide (0.14%) and hexanucleotide (0.02%) (Figure 6A). Based on the distribution of SSR motifs, (A)^n, (G)^n and (T)^n (*n* ≥ 10) were the three predominant types among mononucleotide SSR motifs, with frequencies of 30.93, 30.14 and 27.77%, respectively (Figure 6B). In the six types of dinucleotide repeat motifs, GT (27.93%) was the most common motif, followed by AT (26.34%) and AC (22.2%) (Figure 6C). Among 20 types of trinucleotide repeats, AGC (13.91%), GCT (13.64%) and AGG (11.86%) were the three predominant types (Figure 6D). Tetranucleotide and pentanucleotide SSR motifs contained 35 and 23 types of repeats, respectively (Figures 6E and F).

**Figure 7:**
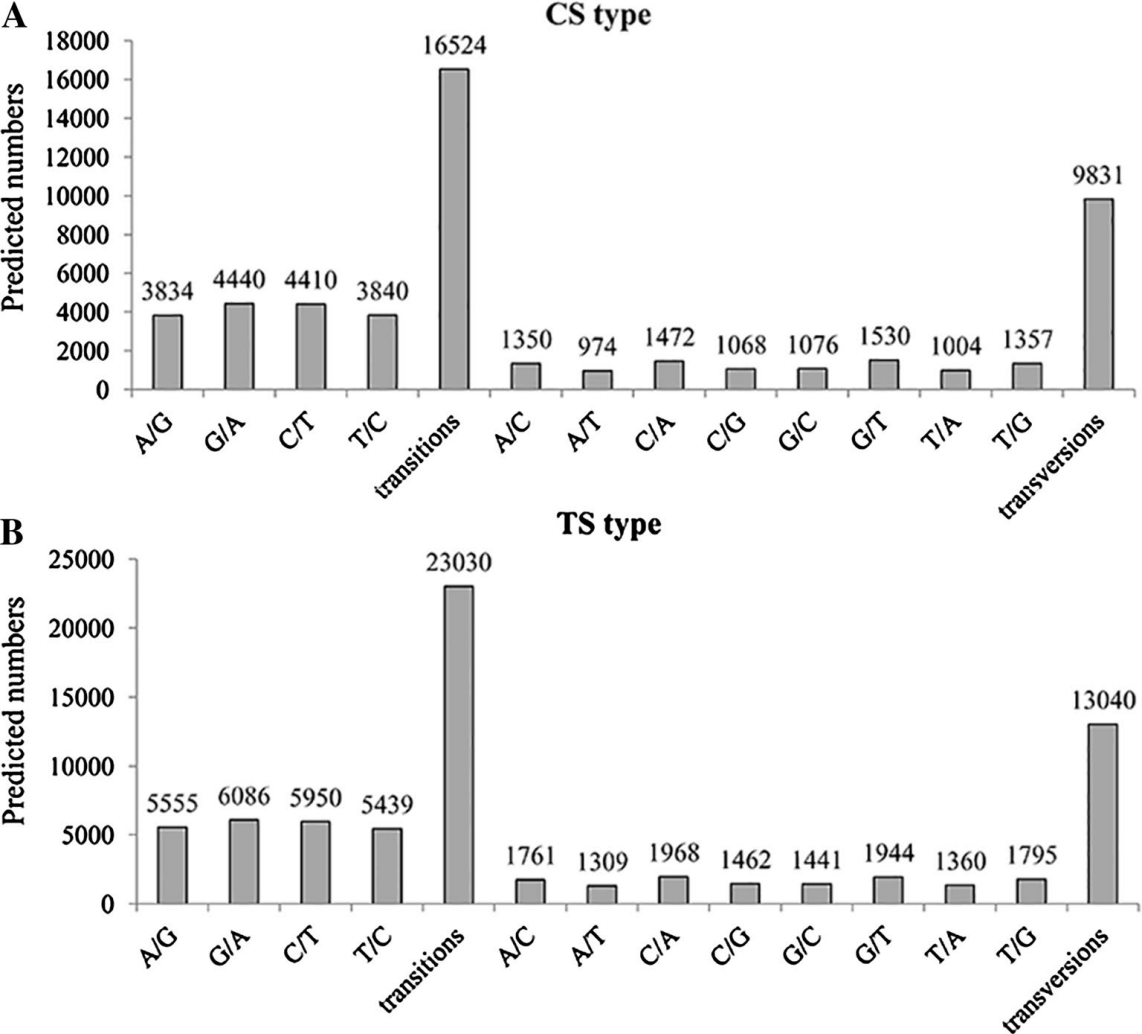
**Distribution of putative single nucleotide polymorphisms (SNP) in the spleen transcriptome.** **A** Distribution of putative SNP in uninfected (CS type) Chinese giant salamanders. **B** Distribution of putative SNP in GSIV-infected (TS type) Chinese giant salamanders.

SNPs were identified from alignments of multiple sequences used for contig assembly. A total of 26 355 and 36 070 SNPs were obtained from the GS_CS and GS_TS samples, respectively. In the GS_CS samples, 16 524 were putative transitions and 9831 were putative transversions (Figure 7A). In the GS_TS samples, the numbers of various SNP types were higher than that in GS_CS samples (Figures 7A and B), with 23 030 SNPs for transitions and 13 040 SNPs for transversions (Figure 7B). No insertion–deletion polymorphisms (indels) were found in either the GS_CS or GS_ TS samples.

### DEGs, SSRs and SNPs analysis related to disorders of nucleotide excision repair

Among the sequencing libraries, we identified 75 sequences related to nucleotide excision repair. The members of the DNA repair gene family including ERCC1-6, ERCC8, XPA, POLH, BLM, TFIIH1-4, RBX1, Cul4, DDB1, DDB2, XPC, HR23, CETN2, CDK7, MNAT1, CCNH, TTDA, RPA, PCNA, RFC and LIG1 were all expressed in the spleen of the Chinese giant salamander. However, 10 genes including BLM, DDB2, ERCC4, ERCC5, ERCC6, ERCC8, POLH, RPA1, TFIIH3 and XPA were DEGs, with 11 isogenes up-regulated and 6 isogenes down-regulated (Table 3).

**Table 3:** DEGs related to disorders of nucleotide excision repair.

Transcript_id	KEGG GENE NAME	CS fpkm	TS fpkm	log2FC1 (TS/CS)	Significant
comp90201_c0_seq1	BLM	1.18	0	−8.73	Yes
comp90201_c0_seq2	BLM	0	0.99	8.29	Yes
comp90773_c0_seq2	DDB2	0	1.18	6.51	Yes
comp93741_c0_seq2	ERCC4, XPF	0	0.25	6.27	Yes
comp92742_c0_seq18	ERCC5, XPG	0.54	0	−7.86	Yes
comp91446_c0_seq12	ERCC6, CSB	0	0.35	6.94	Yes
comp91446_c0_seq15	ERCC6, CSB	0.45	0	−7.57	Yes
comp91446_c0_seq3	ERCC6, CSB	0.57	0	−7.89	Yes
comp91446_c0_seq4	ERCC6, CSB	0	0.61	7.82	Yes
comp91446_c0_seq8	ERCC6, CSB	0	0.63	7.85	Yes
comp81303_c0_seq1	ERCC8, CSA	0	0.59	6.14	Yes
comp81303_c0_seq2	ERCC8, CSA	0.82	0	−6.78	Yes
comp92141_c0_seq1	POLH	0	0.18	5.53	Yes
comp92141_c0_seq3	POLH	0	0.19	5.53	Yes
comp88203_c0_seq1	RPA1, POLR1A	0	3.78	7.76	Yes
comp86879_c0_seq2	TFIIH3, TFB4	1.61	0	−7.53	Yes
comp91081_c0_seq4	XPA	0	1.46	7.99	Yes

DEGs was filtered using threshold of false discovery rate (FDR) ≤0.05 and absolute value of log2Ratio ≥1.

Further analysis of SSRs and SNPs involved in disorders of nucleotide excision repair indicated a total of 12 genes that were related to disorders of nucleotide excision repair that contained 21 SSRs motifs (Table 4). Among them, DEGs including BLM, ERCC6 and ERCC8 contained 1 mononucleotide SRR and 4 trinucleotide SRR motifs. Thirteen genes including ERCC1, ERCC2, CCNH, Cul4, DDB1, LIG1, PCNA, RBX1, RPA, RFC, TFIIH2, TTDA and XPA contained 76 SNP sites (Table 5). Up to 15 SNPs existed in the isogene of PCNA (comp90786_c1_seq5). Among 76 SNPs, 33 (43.42%) and 29 (38.16%) of the SNPs were specific for the uninfected and infected Chinese giant salamanders, respectively. There were 14 SNPs, that were identical for the GS_CS and GS_TS samples but different from the reference sequences.

**Table 4:** SSRs related to disorders of nucleotide excision repair.

Transcript_id	KEGG GENE NAME	Start BP	Repeat	End BP	Type
comp90201_c0_seq1	BLM	5420	(GCT)^4	5432	Trinucleotide
comp90201_c0_seq1	BLM	3386	(ATC)^4	3398	Trinucleotide
comp57681_c0_seq1	CDK7	43	(AT)^6	55	Dinucleotide
comp71102_c0_seq1	CUL4	944	(A)^10	954	Mononucleotide
comp107286_c0_seq1	ERCC3, XPB	2478	(G)^10	2488	Mononucleotide
comp107286_c0_seq1	ERCC3, XPB	2204	(AGC)^4	2216	Trinucleotide
comp93741_c0_seq1	ERCC4, XPF	3807	(T)^10	3817	Mononucleotide
comp91446_c0_seq12	ERCC6, CSB	3738	(AGG)^6	3756	Trinucleotide
comp91446_c0_seq15	ERCC6, CSB	3727	(AGG)^6	3745	Trinucleotide
comp81303_c0_seq1	ERCC8, CSA	66	(T)^14	80	Mononucleotide
comp33205_c0_seq1	RAD23, HR23	1046	(AGC)^4	1058	Trinucleotide
comp33042_c0_seq1	RBX1, ROC1	1	(T)^11	12	Mononucleotide
comp89984_c0_seq1	rpa	577	(G)^10	587	Mononucleotide
comp33075_c0_seq1	RPA2	191	(GGT)^4	203	Trinucleotide
comp57325_c0_seq1	RPA12	1	(G)^21	22	Mononucleotide
comp72565_c0_seq1	RPA3	59	(A)^12	71	Mononucleotide
comp56791_c0_seq1	RFC	1	(T)^13	14	Mononucleotide
comp36422_c0_seq1	RFC	1	(T)^11	12	Mononucleotide
comp37382_c0_seq1	RFC	192	(GGT)^4	204	Trinucleotide
comp53798_c0_seq1	TFIIH1, TFB1	2301	(AC)^7	2315	Dinucleotide
comp82958_c1_seq1	TFIIH2, SSL1	3	(A)^19	22	Mononucleotide

**Table 5:** List of SNPs ralated to disorders of nucleotide excision repair.

Transcript_id	KEGG GENE NAME	Site	Ref	CS_type	TS_type
comp74141_c0_seq1	ERCC1	2223	G	–	A
comp74141_c0_seq2		2070	G	–	A
comp74141_c0_seq2		847	G	T	T
comp88233_c0_seq1	ERCC2	128	G	A	–
comp88233_c0_seq1		2140	T	–	C
comp88233_c0_seq1		2450	G	–	T
comp108677_c0_seq1	CCNH	190	C	T	–
comp61358_c0_seq1	Cul4A	1710	A	C	–
comp61358_c0_seq1		871	C	–	A
comp71102_c0_seq1	Cul4B	1738	A	–	G
comp71102_c0_seq1		811	A	–	G
comp32714_c0_seq1	DDB1	129	G	–	A
comp32717_c0_seq1		1429	G	–	A
comp32717_c0_seq1		433	T	C	–
comp32741_c0_seq1		1157	G	C	C
comp80349_c0_seq1	LIG1	3623	C	–	T
comp90786_c1_seq5	PCNA	105	T	C	C
comp90786_c1_seq5		13	G	–	A
comp90786_c1_seq5		1314	G	–	A
comp90786_c1_seq5		1683	A	G	–
comp90786_c1_seq5		1934	G	A	A
comp90786_c1_seq5		2038	A	G	–
comp90786_c1_seq5		2068	A	G	G
comp90786_c1_seq5		22	A	G	–
comp90786_c1_seq5		2395	A	G	–
comp90786_c1_seq5		2465	G	–	A
comp90786_c1_seq5		2487	G	A	A
comp90786_c1_seq5		2501	A	G	–
comp90786_c1_seq5		2543	G	–	A
comp90786_c1_seq5		2562	A	G	–
comp90786_c1_seq5		93	G	C	–
comp90786_c1_seq7	PCNA	105	T	C	C
comp90786_c1_seq7		1240	T	C	C
comp90786_c1_seq7		1244	C	A	A
comp90786_c1_seq7		13	G	–	A
comp90786_c1_seq7		1818	G	A	A
comp90786_c1_seq7		2077	A	C	C
comp90786_c1_seq7		22	A	G	–
comp90786_c1_seq7		2401	A	–	G
comp90786_c1_seq7		2615	C	–	G
comp90786_c1_seq7		93	G	C	–
comp33042_c0_seq1	RBX1	89	G	T	–
comp89984_c0_seq1	RPA	1679	C	–	T
comp89984_c0_seq1		1829	G	A	–
comp89984_c0_seq1		2071	G	–	A
comp89984_c0_seq1		2600	C	G	–
comp89984_c0_seq1		2645	G	T	–
comp89984_c0_seq1		3312	A	G	–
comp89984_c0_seq1		799	C	T	–
comp89984_c0_seq1		815	G	T	–
comp33075_c0_seq1	RPA2	100	T	C	C
comp57325_c0_seq1	RPA12	385	G	–	C
comp59041_c0_seq1	RPA49	757	G	A	–
comp59041_c0_seq1		864	A	–	G
comp59044_c0_seq1		380	G	–	C
comp72565_c0_seq1	RPA3	475	A	–	G
comp72565_c0_seq1		802	G	A	–
comp88203_c0_seq2	RPA	4663	G	C	–
comp81979_c0_seq1	RPA43	1011	C	–	T
comp81979_c0_seq1		1032	C	T	–
comp81979_c0_seq1		1098	G	–	A
comp56791_c0_seq1	RFC1	3527	T	C	C
comp56791_c0_seq1		3757	A	–	G
comp36422_c0_seq1	RFC2	1188	C	–	T
comp85228_c0_seq1	RFC2	1319	T	G	–
comp85228_c0_seq1		1744	A	C	–
comp85228_c0_seq1		1844	C	T	–
comp36017_c0_seq1	RFC3	335	A	C	C
comp37382_c0_seq1	RFC3	49	C	T	–
comp82958_c1_seq1	TFIIH2	122	G	–	A
comp57951_c0_seq1	TTDA	513	G	T	–
comp91081_c0_seq1	XPA	1705	T	C	–
comp91081_c0_seq3		1633	T	G	–
comp91081_c0_seq3		2827	A	T	–
comp91081_c0_seq4		2669	A	T	–
comp91081_c0_seq4		2893	C	A	A

The identical bases with reference sequences were indicated with “–”.

### DEGs, SSRs and SNPs analysis related to antiviral activity

RIG-I-like receptor and Toll-like receptor signaling pathways are two pivotal pathways involved in antiviral immune responses. In the Chinese giant salamander, 143 isogenes involved in the RIG-I-like receptor signaling pathway and 201 isogenes involved in the Toll-like receptor signaling pathway were identified. However, only 20 and 18 isogenes were DEGs for the RIG-I-like receptor and Toll-like receptor signaling pathways, respectively (Additional file 6). Interestingly, the expression of antiviral pattern recognition receptors such as RIG-I, MDA5, LGP2, TLR3, TLR8, TLR7 and TLR9 did not change in response to the GSIV infection. The expression of NLRX1 was significantly increased, whereas TRIM25 decreased (Additional file 6).

For the RIG-I-like receptor signaling pathway, 21 genes contained 38 SSR motifs. Of these, IL8 contained the most SSRs (6 motifs), followed by IRF7 (5 motifs) and RIG-I (4 motifs). For the Toll-like receptor signaling pathway, 21 genes that included AKT, CCL3,ERK1, FADD, IKBKE,IKBKG, IL8, IRF7, JUN, MAP2K7, MAP3K7IP2, MYD88, NFKB1, NFKBIA, P38, PIK3C, PIK3R, TBK1, TBK1, TLR1 and TLR5 contained 59 SSR motifs. Among them, AKT contained the most mononucleotide SSRs, and PIK3C had the most trinucleotide SSRs (Additional file 7).

For SNPs analysis, 25 genes involved in the RIG-I-like receptor signaling pathway contained 272 motifs. Up to 75 and 52 SNPs existed in the genes of IKBKG and OTUD5, respectively. Among 272 SNPs, 207 (76.1%) SNPs were specific for the GSIV-infected Chinese giant salamanders. The antiviral PRRs MDA5, LGP2 and RIG-I also contained SNP motifs. For the Toll-like receptor signaling pathway, 41 genes contained 445 SNPs that consisted of 141 SNPs (31.69%) specific for the GS_CS samples and 228 SNPs (51.24%) specific for the GS_TS samples, and 73 common SNPs (16.4%) for both the GS_CS and GS_TS samples (Additional file 8). The genes involved in both the RIG-I-like receptor and Toll-like receptor signaling pathways, which included IRF3, IRF7, NFKBIA, FADD, JNK, RELA, IL12B, IL8, CASP8, P38, IKBKA, IKBKE and IKBKG contained a total of 141 SNPs (Additional files 8 and 9).

### Experimental validation

**Figure 8:**
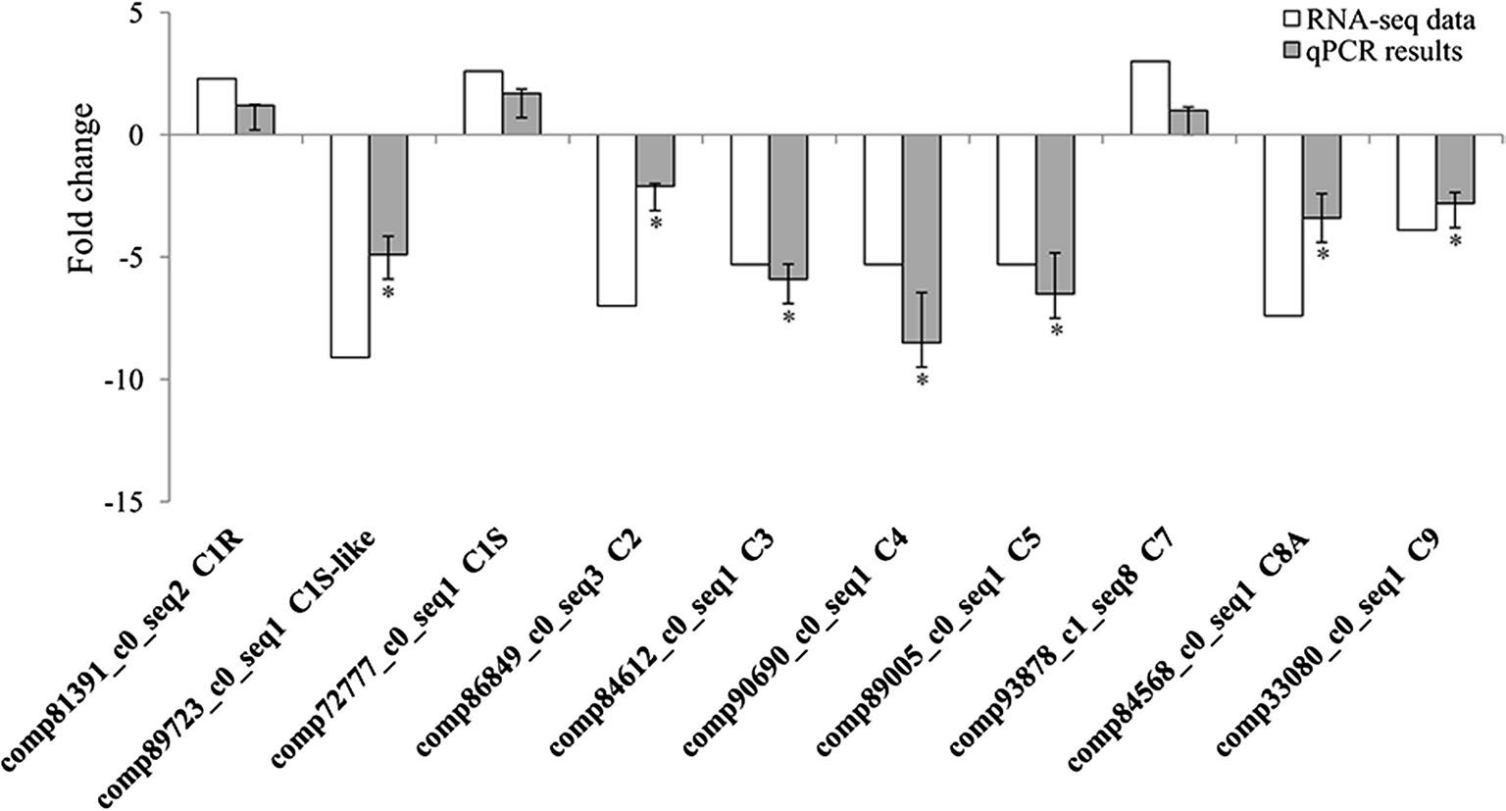
**Expression pattern validation of selected genes by qPCR.** White bar indicates transcript abundance changes calculated by the RPKM method. Black bar with associated standard error bar represents relative expression level determined by qPCR using 2^−ΔΔCT^ method. Results represent mean standard deviations (±SD) of three experimental replicates.

Since the most enriched pathway was the “complement and coagulation cascades”, 10 genes belonging to the complement components were selected and used for qPCR validation. PCR amplification showed that all qPCR primers produced single fragments of the expected lengths (141–354 bp). Except for complement components C1R (comp81391_c0_seq2), C1S (comp72777_c0_seq1) and C7 (comp93878_c1_seq8), the expression of other 7 complement components including C1S-like, C2, C3, C4, C5, C8A and C9 was in agreement with their transcript abundance changes determined by RNA-seq (Figure 8).

## Discussion

### Transcriptome sequencing of spleen samples from uninfected and infected Chinese giant salamanders

The complete genome of GSIV has been sequenced [12]. However, the available molecular information is limited for the Chinese giant salamander, and not sufficient for the investigation of the mechanisms of immune reactivity against pathogen infection. In recent years, a number of reports reveal that the transcriptome sequencing of cDNA is an efficient method for generating large sequences that represent expressed genes [33]. Given that the spleen is one of the most important immune organs and is also the main target organ for the iridovirus GSIV [10, 22, 34, 35], the transcriptome sequencing of spleen samples from uninfected and infected Chinese giant salamanders was expected to provide abundant ESTs for amphibian immune genes and contribute to the understanding of GSIV-host interactions. In the study reported herein, using the recently developed Solexa sequencing technology and Trinity RNA-Seq assembly, the results of transcriptome sequencing are reported for the Chinese giant salamanders.

### TLR, NLR and the RLR systems that are involved in immune responses

To begin this line of research, we first checked a set of sequences that encode components of TLR, NLR and the RLR systems that are involved in immune responses. In the spleen transcriptome of the Chinese giant salamanders, a total of 201, 206 and 143 isogenes were identified to be involved in the TLR, NLR and RLR pathways, respectively. We used MEGA software to compare the encoded proteins for these three pathways from the Chinese giant salamanders with their mammalian and teleost counterparts. For some proteins, the annotated sequences were not complete, therefore the phylogenetic trees constructed show relationships, but do not show precise evolutionary distances. The conservation and divergence of the TLR, NLR and RLR families are summarized below and in the figure legends.

#### The TLR system

The Toll-like Receptors (TLRs) are present throughout virtually the entire animal kingdom and have important functions in initiating inflammatory responses and shaping adaptive immunity [36]. A typical TLR is a type I transmembrane protein with many extracellular leucine-rich repeat (LRR) motifs for ligand recognition, and a cytoplasmic TIR domain for signal transduction. In invertebrates, only one or two TLR genes exist in sea squirt *Cionaintestinalis* and the nematode *Caenorhabditiselegans* [37, 38], however the sea urchin *Strongylocentrotus purpuratus* and amphioxus *Branchiostoma lanceolatum* possess a large number of TLRs [39, 40]. In mammals, the human genome contains 10 functional TLRs whereas the mouse genome contains 12 TLRs, with TLR10 being a pseudogene, and TLR11, TLR12 and TLR13 being mouse-specific genes [41]. Most fish species possess a higher number of TLR genes than mammalian species due to the presence of duplicated TLRs and fish-specific TLRs [42, 43].

**Figure 9:**
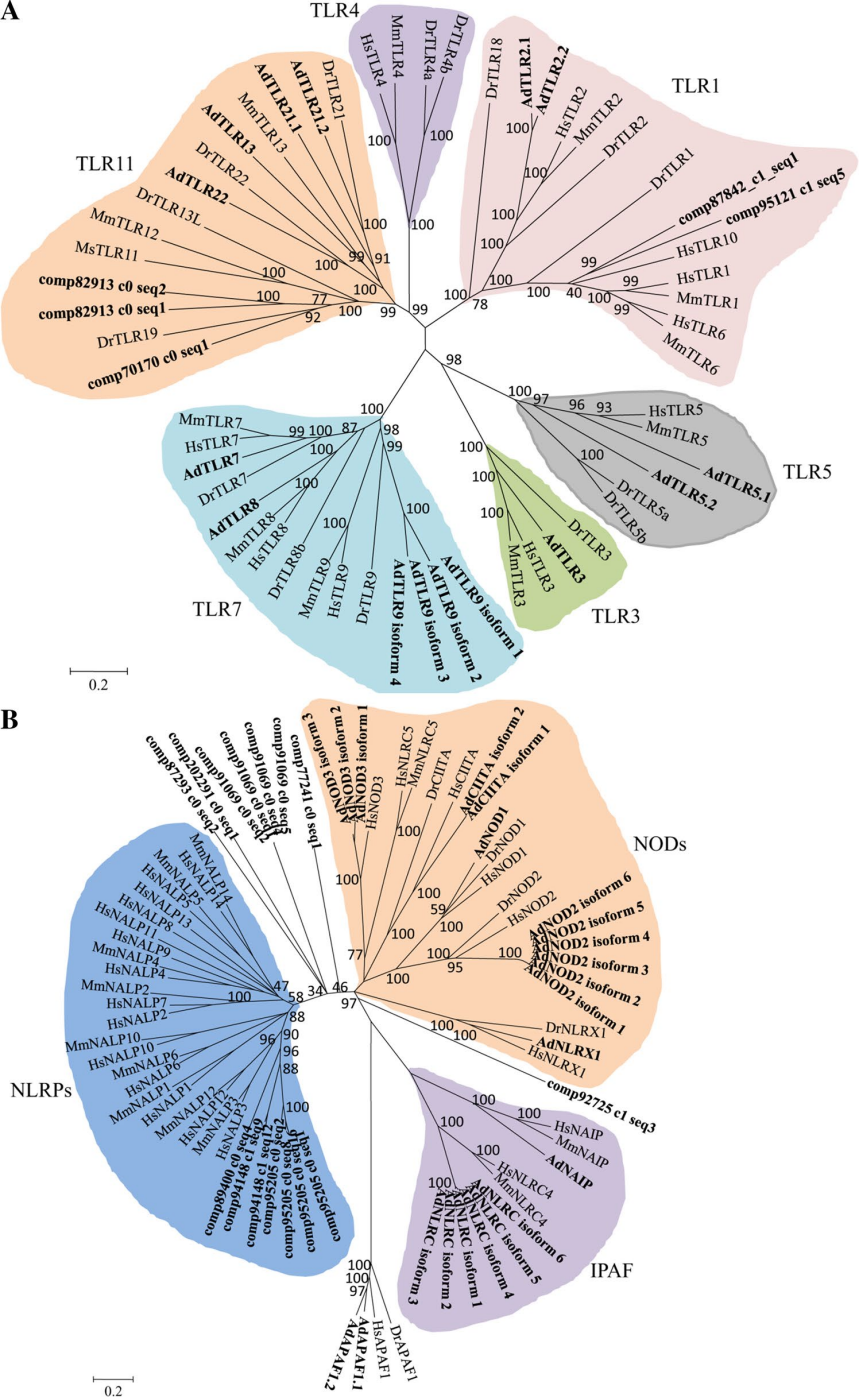
**Phylogenetic tree of Toll-like receptors (A) and NACHT-domain family (B).** Phylogenetic relationships were based on amino acid alignments. Bootstrap values based on 10 000 replicates are indicated on each branch. The evolutionary history was inferred using the neighbor-joining method. All positions containing gaps and missing data were eliminated from the dataset (pairwise deletion). Accession numbers of sequences used to build the tree are presented in Additional file 11.

Six major TLR families were identified in all vertebrate taxa designated as TLR1, TLR3, TLR4, TLR5, TLR7 and TLR11 [44]. In the Chinese giant salamanders, the members of TLR3, TLR5 and TLR7 have clear orthologs with fish and mammals that was supported by the phylogenetic analysis (Figure 9A). For TLR1 family, clear orthologous relationships were found among fish, amphibians and mammals for TLR2 gene, and two sequences were found to group with mammalian TLR10, TLR1 and TLR6. Interestingly, the ortholog of the mouse-specific TLR13 and fish-specific TLR21 and 22 appear to exist in the Chinese giant salamander. Similar to fish, the Chinese giant salamander has duplicated copies of some TLRs, such as TLR2, TLR5 and TLR21. These results suggest that amphibians may have a greater TLR repertoire than fish or mammals as a result of living in both aquatic and terrestrial environments.

#### The NLR system

A large family of cytoplasmic NACHT-LRR receptors (NLRs), characterized by the presence of a nucleotide-binding protein (NACHT domain), have important functions in apoptosis, inflammation and innate immune signaling [45–48]. There are 20–30 NLR genes in vertebrates, which are divided into three distinct subfamilies with regard to their phylogenetic relationships including the NODs (NOD1-2, NOD3/NLRC3, NOD4/NLRC5, NOD5/NLRX1, CIITA), the NLRPs (NLRP1–14, also called NALPs) and the IPAF subfamily, consisting of IPAF (NLRC4) and NAIP [49]. In the Chinese giant salamander, the canonical NOD proteins (NOD1, NOD2, NOD3, NOD5, CIITA), IPAF, NAIP, as well as APAF1 (apoptotic protease activating factor 1) exhibit clear orthologous relationships (Figure 9B). However, no homologous sequences were found for NOD4 in the spleen transcriptome of the Chinese giant salamander.

The expansion of NLR-encoding genes has been described in the sea urchin, amphioxus and zebrafish [39, 40, 50, 51]. In their genome, at least 92 (amphioxus) and 200 (sea urchin and zebrafish) NLR genes were predicted. The large majority of the sea urchin NLR proteins consist of a central NACHT domain, an N-terminal DEATH domain and C-terminal LRRs [39]. However, the large groups of fish-specific NLR proteins do not contain any the amino-terminal effector domains [51]. Additionally, NLRP proteins with a PYD domain in the N-terminal region were not found in the sea urchin, amphioxus and zebrafish [39, 40, 51]. Unlike NLRs in the species mentioned above, the expansion of NLR proteins were not found in the spleen transcriptome of the Chinese giant salamander, however many annotated sequences grouped with the mammalian NLRPs in the phylogenetic tree (Figure 9B).

In the Chinese giant salamander, many NLRs such as APAF1, IPAF, NOD2, NOD3, CIITA and NALP3/NALP12 existed as multiple isoforms. Our previous studies have shown that the isoforms of immune genes regulated positively or negatively antibacterial and antiviral immunity [52–54]. The multiple isoforms of NLR genes in the present study suggested that the complexity and diversity of the innate immunity may be achieved in Chinese giant salamander through the use of alternative splicing or gene duplication.

#### The RLR system

Besides NLRs, the RIG-I-like receptors (RLRs) represent another crucial family of intracellular pattern recognition receptors, which use C-terminal RNA helicases to recognize viral RNA and N-terminal CARD domains for signaling [55]. The RLR family members include retinoic acid inducible gene-I (RIG-I), melanoma differentiation gene-5 (MDA5) and laboratory of genetics and physiology-2 (LGP2), which have been identified in teleost species [52, 56–59]. These three RLRs were present in the Chinese giant salamander. As for adapters, the CARD-containing IPS-1 gene wasn’t identified in the spleen transcriptome of the Chinese giant salamander (Additional file 10A).

### Comparative transcriptome analysis revealed the immune anergy of TLR, NLR and the RLR systems in the spleen

Iridoviruses are large double-stranded DNA (dsDNA) viruses that can infect invertebrates and poikilothermic vertebrates, including insects, fish, amphibians and reptiles. A real-time polymerase chain reaction (PCR) assay for a marine fish iridovirus showed that the spleen and kidney contained the largest number of viral particles while no viral DNA was detected in the muscle tissue [34]. A comparison of the genes expressed during a red seabream iridovirus (RSIV) infection in the spleen and kidney suggested that RSIV preferentially targets the spleen [36]. In the Chinese giant salamander, our previous study showed tissue necrosis and the existence of GSIV viral particles in the spleen, liver and kidney [10]. Many studies have shown that the spleen and kidney are the target organs of iridovirus infections.

Using 454 pyrosequencing, 755 up-regulated genes and 695 down-regulated genes were identified in the two spleen-complementary DNA libraries, that were constructed from Singapore grouper iridovirus (SGIV) infected and control orange-spotted grouper [22]. Although 80 367 genes were identified in the spleen transcriptome of the Chinese giant salamander using Solexa sequencing technology, comparative transcriptome analysis indicated that only 293 genes were down-regulated and 220 genes were up-regulated in response to the GSIV infection. In addition, a large number of genes were involved in TLR (72 hits), NLR (63 hits) and RLR (44 hits) pathways, however only 11 (TLR), 12 (NLR) and 10 (RLR) genes in these pathways showed significant changes in their transcripts after GSIV infection. The non-significant changes for the majority of the genes including PRRs and the non-significant enrich for these pathways suggested that TLR, NLR and RLR systems in the spleen of Chinese giant salamanders were immune anergic during GSIV infection.

### Comparative transcriptome analysis revealed the primary immunodeficiency of the complement system in the spleen

The vertebrate complement system is a humoral and proteolytic system that is composed of approximately 40 soluble and membrane-bound proteins. It is an integral part of the innate immune system protecting the host against invasion and proliferation of various pathogens. The mammalian complement system has three different activation pathways which include classical, alternative and lectin. Activation of the classical pathway is triggered by the binding of C1q proteins to immune complexes or aggregates containing IgG or IgM [60]. The lectin pathway parallels the classical pathway, the difference being at the initial step of target recognition and subsequent activation [61]. Activation of the lectin pathway occurs through the binding of the mannose-binding lectins (MBL) to their target, which results in the activation of the MBL-associated serine proteases (MASPs) [62]. Different from the classical and lectin pathways, the alternative pathway of complement activation is triggered spontaneously, and primarily makes use of the recognition of host-associated molecular patterns (HAMPs), not pathogen-associated molecular patterns (PAMPs) [63].

In the study reported herein, the majority of genes involved in the complement and coagulation cascades (53 of 59 total genes or 90%) were identified, and the alternative, lectin and classical pathways appeared conserved in the Chinese giant salamander (Additional file 10B). The KEGG enrichment analysis indicated that the most enriched pathway is the “complement and coagulation cascades” and significantly enriched diseases include “primary immunodeficiency” and “complement regulatory protein defects”. In mammals, primary immunodeficiencies (PIDs) are severe defects in the capacity of the host to mount a proper immune response and are characterized by an increased susceptibility to infections. Common PIDs include disorders of humoral immunity, T-cell defects, combined B- and T-cell defects, phagocytic disorders, and complement deficiencies [64]. In this study, the different genes that were expressed in the “primary immunodeficiency” were mainly the components of complement system, with down-regulated expression of C1s-like, C2, C3, C4, c8g.1, C9, complement component (3b/4b) receptor 1-like, complement receptor type 2-like, complement factor H and hemolytic complement. The data from the qRT-PCR analysis also validated the down-regulated expression of the complement components C1s-like, C2, C3, C4, C5, C8A and C9 in response to the GSIV infection, however the weak up-regulated expression of C1r, C1s and C7 in the transcriptome analysis could not be validated by qRT-PCR. The results supported that GSIV infection can trigger primary immunodeficiency of the complement system in the spleen of the Chinese giant salamander.

## Additional files

10.1186/s13567-015-0279-8 **Oligonucleotide primers used for expression analysis.** The table listed the sequences of all primers used in Quantitative real-time PCR for gene expression validation.
10.1186/s13567-015-0279-8 **KEGG summary of Chinese giant salamander isogenes.** A total of 23 712 isogenes were assigned to 317 KEGG pathways. For each pathway, the pathway definition, number of sequences and isogene ID were listed in the table.
10.1186/s13567-015-0279-8 **Differentially expressed genes specific for uninfected Chinese giant salamander (A) and GSIV-infected Chinese giant salamander (B).** The table provided a comprehensive list of differentially expressed genes specific for uninfected Chinese giant salamander (GS_CS) and GSIV-infected Chinese giant salamander (GS_TS) at estimated absolute log2-fold change of ≥1 and FDR ≤ 0.05. A total of 102 down-regulated genes were detected only in the GS_CS samples (A), and 67 up-regulated genes specific for the GS_TS samples (B).
10.1186/s13567-015-0279-8 **GO functional enrichment analysis of differentially expressed genes between uninfected and GSIV-infected Chinese giant salamander.** This additional file showed that 2493 differentially expressed genes (DEGs) were enriched in 433 GO terms. The GO terms showed significant enrichment of “immune system process” and “response to stimulus” in addition to the other cellular processes. The GO terms with a corrected *P* value ≤ 0.05 were defined as significantly enriched in DEGs.
10.1186/s13567-015-0279-8 **Enrichment analysis of KEGG PATHWAY and KEGG DISEASE.** This additional file showed that differentially expressed genes were enriched in 7 KEGG pathways and 14 KEGG diseases.
10.1186/s13567-015-0279-8 **DEGs related to antiviral signaling pathway.** This file showed that 20 and 18 isogenes were differentially expressed genes for the RIG-I-like receptor and Toll-like receptor signaling pathways, respectively.
10.1186/s13567-015-0279-8 **SSRs related to antiviral signaling pathway.** The file provided a list of SSRs related to the RIG-I-like receptor and the Toll-like receptor signaling pathways. The SSR items including repeat, type, start and end positions were listed in the table.
10.1186/s13567-015-0279-8 **SNPs analysis related to antiviral signaling pathway.** This additional table provided a detail of SNPs involved in RIG-I-like receptor and the Toll-like receptor signaling pathways. The number of SNPs, which appeared in GSIV-infected (TS), uninfected (CS) and both of TS and CS samples, were also listed.
10.1186/s13567-015-0279-8 **A list of SNPs related to the RIG-I-like receptor signaling pathway (A) and the Toll-like receptor signaling pathway (B).** 272 and 442 SNPs related to the RIG-I-like receptor and the Toll-like receptor signaling pathways, respectively, were listed in this additional file. The identical bases with reference sequences were indicated with “–”.
10.1186/s13567-015-0279-8 **ESTs in the spleen library of Chinese giant salamanders hit to the RIG-I-like receptor signaling pathway (A) and complement and coagulation cascades (B).** 44 ESTs including RIG-I, MDA5 and LGP2 were identified in the spleen library of Chinese giant salamanders, which hit to the RIG-I-like receptor signaling pathway (A). The majority of genes involved in the complement and coagulation cascades (53 of 59 total genes or 90%) were identified, and the alternative, lectin and classical pathways appeared conserved in the Chinese giant salamander (B).
10.1186/s13567-015-0279-8 **Accession numbers of sequences used to build the phylogenetic tree.** A total of 78 genes, which were used to build the phylogenetic tree, and their accession numbers were listed in this additional file.
