# Supplementary material for: Transcriptomic analysis of the host response to an iridovirus infection in Chinese giant salamander, Andrias davidianus
Source: Vet Res. 2015 Nov 20;46:136. doi: 10.1186/s13567-015-0279-8 (PMC4654921; doi:10.1186/s13567-015-0279-8)
Supplement: Supplementary file 1 — 10.1186/s13567-015-0279-8 Oligonucleotide primers used for expression analysis. The table listed the sequences of all primers used in Quantitative real-time PCR for gene expression validation. [file 13567_2015_279_MOESM1_ESM.docx]

**Additional file 10 Oligonucleotide primers used for expression analysis**

| **Primer name** | **Primer sequence** | **Size (bp)** |
| --- | --- | --- |
| AdactinF | AAGGTTATGCCCTGCCTCACG | 184 |
| AdactinR | GCCATTTCCTGCTCAAAGTCC |  |
| AdC1RF | GGACGGGAAATCGGAAACTA | 216 |
| AdC1RR | GTCCTGGAAGCGGAAATCTG |  |
| AdC1SF | GCAAGGATATTTGGAGGCG | 267 |
| AdC1SR | GTTGTAATCGGTGCGAGTGTT |  |
| AdC1S-likeF | TAGAATTTGCCTATGCGGGTTTA | 294 |
| AdC1S-likeR | GTATCCTTGCCTTCCTGGTGTTA |  |
| AdC2F | TACCTCCAGAACTACGGCGACTTAC | 344 |
| AdC2R | ACAATGACTTGCTCCACGTCCACTT |  |
| AdC3F | CAGGTGGTGATTATGTCCG | 296 |
| AdC3R | CTCCAGAAGGGTGTTGTCC |  |
| AdC4F | ACACGGCAGTACCAGAGTGCGTAGAAT | 176 |
| AdC4R | AACACTTCCTTGGCACAATGCTGATAG |  |
| AdC5F | TGAGACCCTTCCAGCGTTATGAGC | 204 |
| AdC5R | TCCTGTCTTTCCTTCGCCCAATCT |  |
| AdC7F | AGTGCGTCTGTGGAGTGGGTAA | 193 |
| AdC7R | CAATGAAGCGTGAAATGTCTGTT |  |
| AdC8AF | TGACTTCACGAACAGGCACCAACG | 141 |
| AdC8AR | TTCCAATACACCAAGCTCCACCATCT |  |
| AdC9F | GCTGGGCATCTTCCTTGGT | 354 |
| AdC9R | GGGTACGGCTCCGATTCTTAT |  |
