# Supplementary material for: Transcriptomic analysis of the host response to an iridovirus infection in Chinese giant salamander, Andrias davidianus
Source: Vet Res. 2015 Nov 20;46:136. doi: 10.1186/s13567-015-0279-8 (PMC4654921; doi:10.1186/s13567-015-0279-8)
Supplement: Supplementary file 6 — 10.1186/s13567-015-0279-8 DEGs related to antiviral signaling pathway. This file showed that 20 and 18 isogenes were differentially expressed genes for the RIG-I-like receptor and Toll-like receptor signaling pathways, respectively. [file 13567_2015_279_MOESM6_ESM.docx]

**Additional file 7 DEGs related to antiviral signaling pathway***

| **Transcript_id** | **KEGG GENE NAME** | **CS fpkm** | **TS fpkm** | **log2FC1**  **(TS/ CS)** | **significant** |
| --- | --- | --- | --- | --- | --- |
| **RIG-I-like receptor signaling pathway** | | | | | |
| comp94535_c0_seq1 | CYLD, USLP2 | 0.55 | 0 | -6.65 | yes |
| comp94126_c0_seq1 | IKBKG, IKKG | 0 | 1.56 | 7.91 | yes |
| comp94126_c0_seq6 | IKBKG, IKKG | 0 | 3.14 | 8.87 | yes |
| comp78002_c0_seq1 | IL12B | 0.27 | 6.4 | 4.33 | yes |
| comp59200_c0_seq1 | IL8, CXCL8 | 4.97 | 31.11 | 2.44 | yes |
| comp83245_c0_seq1 | IL8, CXCL8 | 1.53 | 12.25 | 2.79 | yes |
| comp91257_c1_seq1 | IL8, CXCL8 | 0 | 3.57 | 7.93 | yes |
| comp88607_c0_seq2 | NLRX1 | 0 | 1.77 | 8.32 | yes |
| comp94009_c0_seq1 | OTUD5, DUBA | 0.24 | 1.97 | 2.85 | yes |
| comp94009_c0_seq5 | OTUD5, DUBA | 0 | 0.29 | 5.41 | yes |
| comp93194_c3_seq2 | P38 | 1.57 | 0 | -8.08 | yes |
| comp93194_c3_seq3 | P38 | 0 | 3.6 | 9.14 | yes |
| comp90397_c0_seq1 | TANK | 0 | 0.82 | 7.50 | yes |
| comp90397_c0_seq7 | TANK | 0 | 0.59 | 6.14 | yes |
| comp94100_c0_seq10 | TRAF2 | 0 | 0.21 | 6.40 | yes |
| comp94100_c0_seq2 | TRAF2 | 0 | 0.13 | 5.73 | yes |
| comp94100_c0_seq3 | TRAF2 | 0 | 0.42 | 7.36 | yes |
| comp94100_c0_seq8 | TRAF2 | 0 | 0.12 | 5.63 | yes |
| comp94100_c0_seq9 | TRAF2 | 0 | 0.64 | 7.97 | yes |
| comp90705_c0_seq3 | TRIM25, EFP | 0.79 | 0.03 | -4.42 | yes |
| **Toll-like receptor signaling pathway** | | | | | |
| comp32948_c0_seq1 | CXCL9 | 22.97 | 480.63 | 4.186099 | yes |
| comp84557_c0_seq1 | FOS | 0.04 | 2.11 | 5.433759 | yes |
| comp94126_c0_seq1 | IKBKG, IKKG | 0 | 1.56 | 7.909248 | yes |
| comp94126_c0_seq6 | IKBKG, IKKG | 0 | 3.14 | 8.874602 | yes |
| comp78002_c0_seq1 | IL12B | 0.27 | 6.4 | 4.326182 | yes |
| comp91257_c1_seq1 | IL8, CXCL8 | 0 | 3.57 | 7.929921 | yes |
| comp83245_c0_seq1 | IL8, CXCL8 | 1.53 | 12.25 | 2.785526 | yes |
| comp59200_c0_seq1 | IL8, CXCL8 | 4.97 | 31.11 | 2.443848 | yes |
| comp94759_c0_seq10 | IRAK1 | 0 | 0.35 | 5.631465 | yes |
| comp94759_c0_seq1 | IRAK1 | 0 | 0.26 | 5.526787 | yes |
| comp84662_c1_seq3 | MAP3K7IP1 | 0.3 | 2.88 | 3.136021 | yes |
| comp93194_c3_seq3 | P38 | 0 | 3.6 | 9.137124 | yes |
| comp93194_c3_seq2 | P38 | 1.57 | 0 | -8.07767 | yes |
| comp77558_c0_seq2 | PIK3C | 0.33 | 0 | -6.42623 | yes |
| comp89291_c0_seq1 | TIRAP | 0 | 0.72 | 6.762258 | yes |
| comp89291_c0_seq2 | TIRAP | 1.03 | 0 | -7.49537 | yes |
| comp87842_c1_seq1 | TLR1 | 0 | 0.42 | 5.987553 | yes |
| comp87842_c1_seq3 | TLR1 | 0 | 0.37 | 5.631465 | yes |

* DEGs was filtered using threshold of false discovery rate (FDR) ≤ 0.05 and absolute value of log2Ratio ≥ 1.
