# Supplementary material for: Transcriptomic analysis of the host response to an iridovirus infection in Chinese giant salamander, Andrias davidianus
Source: Vet Res. 2015 Nov 20;46:136. doi: 10.1186/s13567-015-0279-8 (PMC4654921; doi:10.1186/s13567-015-0279-8)
Supplement: Supplementary file 7 — 10.1186/s13567-015-0279-8 SSRs related to antiviral signaling pathway. The file provided a list of SSRs related to the RIG-I-like receptor and the Toll-like receptor signaling pathways. The SSR items including repeat, type, start and end positions were listed in the table. [file 13567_2015_279_MOESM7_ESM.docx]

**Additional file 8 SSRs related to antiviral signaling pathway**

| **Transcript_id** | **KEGG GENE NAME** | **Start BP** | **Repeat** | **End BP** | **Type** |
| --- | --- | --- | --- | --- | --- |
| **RIG-I-like receptor signaling pathway** | | | | | |
| comp59161_c0_seq1 | AZI2 | 1717 | (A)^16 | 1733 | Mononucleotide |
| comp94037_c0_seq1 | CASP10 | 999 | (C)^11 | 1010 | Mononucleotide |
| comp94037_c0_seq1 | CASP10 | 1519 | (A)^11 | 1530 | Mononucleotide |
| comp85187_c0_seq1 | DAK | 621 | (AC)^6 | 633 | Dinucleotide |
| comp57789_c0_seq1 | DDX3X, bel | 2249 | (AGC)^4 | 2261 | Trinucleotide |
| comp91539_c0_seq1 | DDX58, RIG-I | 9112 | (G)^16 | 9128 | Mononucleotide |
| comp91539_c0_seq1 | DDX58, RIG-I | 8078 | (T)^11 | 8089 | Mononucleotide |
| comp91539_c0_seq1 | DDX58, RIG-I | 7930 | (T)^10 | 7940 | Mononucleotide |
| comp91539_c0_seq1 | DDX58, RIG-I | 7325 | (C)^11 | 7336 | Mononucleotide |
| comp57091_c0_seq1 | FADD | 1065 | (A)^10 | 1075 | Mononucleotide |
| comp85834_c0_seq1 | IKBKE, IKKE | 2704 | (G)^11 | 2715 | Mononucleotide |
| comp94126_c0_seq1 | IKBKG, IKKG | 447 | (G)^10 | 457 | Mononucleotide |
| comp33803_c0_seq1 | IL8, CXCL8 | 1548 | (GAGT)^6 | 1572 | Tetranucleotide |
| comp59200_c0_seq1 | IL8, CXCL8 | 1052 | (AAC)^5 | 1067 | Trinucleotide |
| comp59200_c0_seq1 | IL8, CXCL8 | 1320 | (A)^11 | 1331 | Mononucleotide |
| comp91257_c1_seq1 | IL8, CXCL8 | 507 | (G)^11 | 518 | Mononucleotide |
| comp98544_c0_seq1 | IL8, CXCL8 | 219 | (A)^11 | 230 | Mononucleotide |
| comp98544_c0_seq1 | IL8, CXCL8 | 855 | (AT)^6 | 867 | Dinucleotide |
| comp75390_c1_seq1 | IRF7 | 1 | (AC)^11 | 23 | Dinucleotide |
| comp75390_c1_seq1 | IRF7 | 535 | (A)^13 | 548 | Mononucleotide |
| comp75390_c1_seq1 | IRF7 | 2721 | (G)^10 | 2731 | Mononucleotide |
| comp75390_c1_seq1 | IRF7 | 1214 | (G)^12 | 1226 | Mononucleotide |
| comp88225_c0_seq1 | IRF7 | 2 | (ACC)^4 | 14 | Trinucleotide |
| comp92425_c0_seq1 | ISG15 | 1503 | (G)^14 | 1517 | Mononucleotide |
| comp79747_c0_seq1 | MAP3K1, MEKK1 | 651 | (T)^15 | 666 | Mononucleotide |
| comp93708_c1_seq1 | NFKB1 | 2636 | (ATC)^5 | 2651 | Trinucleotide |
| comp79133_c0_seq1 | NFKBIA | 324 | (A)^10 | 334 | Mononucleotide |
| comp79133_c0_seq1 | NFKBIA | 602 | (C)^10 | 612 | Mononucleotide |
| comp94009_c0_seq1 | OTUD5, DUBA | 2068 | (A)^13 | 2081 | Mononucleotide |
| comp94009_c0_seq1 | OTUD5, DUBA | 2316 | (A)^11 | 2327 | Mononucleotide |
| comp72310_c1_seq1 | P38 | 1 | (A)^15 | 16 | Mononucleotide |
| comp72310_c1_seq1 | P38 | 1521 | (G)^10 | 1531 | Mononucleotide |
| comp86117_c0_seq1 | PIN1 | 177 | (A)^12 | 189 | Mononucleotide |
| comp33180_c0_seq1 | SIKE | 1 | (T)^20 | 21 | Mononucleotide |
| comp33543_c0_seq1 | SIKE | 1 | (T)^11 | 12 | Mononucleotide |
| comp45723_c0_seq1 | TBK1 | 33 | (ATT)^4 | 45 | Trinucleotide |
| comp94100_c0_seq10 | TRAF2 | 6686 | (T)^14 | 6700 | Mononucleotide |
| comp94514_c0_seq10 | TRIM25, EFP | 3396 | (GT)^7 | 3410 | Dinucleotide |
| **Toll-like receptor signaling pathway** | | | | | |
| comp83434_c0_seq1 | AKT | 944 | (T)^11 | 955 | Mononucleotide |
| comp83434_c0_seq1 | AKT | 310 | (T)^10 | 320 | Mononucleotide |
| comp84334_c0_seq1 | AKT | 1773 | (C)^11 | 1784 | Mononucleotide |
| comp88061_c0_seq1 | AKT | 6 | (A)^17 | 23 | Mononucleotide |
| comp88061_c0_seq1 | AKT | 364 | (AT)^7 | 378 | Dinucleotide |
| comp88061_c0_seq1 | AKT | 4402 | (T)^11 | 4413 | Mononucleotide |
| comp88061_c0_seq1 | AKT | 3170 | (G)^10 | 3180 | Mononucleotide |
| comp91452_c0_seq1 | AKT | 3284 | (C)^12 | 3296 | Mononucleotide |
| comp33571_c0_seq1 | CCL3 | 1 | (T)^10 | 11 | Mononucleotide |
| comp98395_c0_seq1 | CCL5 | 558 | (C)^11 | 569 | Mononucleotide |
| comp32948_c0_seq1 | CXCL9 | 988 | (A)^11 | 999 | Mononucleotide |
| comp72268_c2_seq1 | ERK1_2 | 2371 | (G)^11 | 2382 | Mononucleotide |
| comp72268_c2_seq1 | ERK1_2 | 2105 | (GGT)^4 | 2117 | Trinucleotide |
| comp72268_c2_seq1 | ERK1_2 | 1481 | (A)^12 | 1493 | Mononucleotide |
| comp72268_c2_seq1 | ERK1_2 | 2266 | (C)^12 | 2278 | Mononucleotide |
| comp72268_c2_seq1 | ERK1_2 | 2541 | (C)^17 | 2558 | Mononucleotide |
| comp78120_c0_seq1 | ERK1_2 | 1694 | (T)^10 | 1704 | Mononucleotide |
| comp78120_c0_seq1 | ERK1_2 | 1495 | (T)^12 | 1507 | Mononucleotide |
| comp57091_c0_seq1 | FADD | 1065 | (A)^10 | 1075 | Mononucleotide |
| comp85834_c0_seq1 | IKBKE, IKKE | 2704 | (G)^11 | 2715 | Mononucleotide |
| comp94126_c0_seq1 | IKBKG, IKKG | 447 | (G)^10 | 457 | Mononucleotide |
| comp33803_c0_seq1 | IL8, CXCL8 | 1548 | (GAGT)^6 | 1572 | Tetranucleotide |
| comp59200_c0_seq1 | IL8, CXCL8 | 1052 | (AAC)^5 | 1067 | Trinucleotide |
| comp59200_c0_seq1 | IL8, CXCL8 | 1320 | (A)^11 | 1331 | Mononucleotide |
| comp91257_c1_seq1 | IL8, CXCL8 | 507 | (G)^11 | 518 | Mononucleotide |
| comp98544_c0_seq1 | IL8, CXCL8 | 219 | (A)^11 | 230 | Mononucleotide |
| comp98544_c0_seq1 | IL8, CXCL8 | 855 | (AT)^6 | 867 | Dinucleotide |
| comp75390_c1_seq1 | IRF7 | 1 | (AC)^11 | 23 | Dinucleotide |
| comp75390_c1_seq1 | IRF7 | 535 | (A)^13 | 548 | Mononucleotide |
| comp75390_c1_seq1 | IRF7 | 2721 | (G)^10 | 2731 | Mononucleotide |
| comp75390_c1_seq1 | IRF7 | 1214 | (G)^12 | 1226 | Mononucleotide |
| comp88225_c0_seq1 | IRF7 | 2 | (ACC)^4 | 14 | Trinucleotide |
| comp36523_c0_seq1 | JUN | 643 | (C)^10 | 653 | Mononucleotide |
| comp378893_c0_seq1 | MAP2K7, MKK7 | 2226 | (T)^11 | 2237 | Mononucleotide |
| comp73696_c0_seq1 | MAP3K7IP2, TAB2 | 3982 | (GTT)^4 | 3994 | Trinucleotide |
| comp73696_c0_seq1 | MAP3K7IP2, TAB2 | 1606 | (A)^15 | 1621 | Mononucleotide |
| comp91877_c0_seq1 | MAP3K8, COT | 3236 | (G)^10 | 3246 | Mononucleotide |
| comp91877_c0_seq1 | MAP3K8, COT | 3226 | (T)^10 | 3236 | Mononucleotide |
| comp100670_c0_seq1 | MYD88 | 1389 | (T)^12 | 1401 | Mononucleotide |
| comp100670_c0_seq1 | MYD88 | 93 | (A)^10 | 103 | Mononucleotide |
| comp93708_c1_seq1 | NFKB1 | 2636 | (ATC)^5 | 2651 | Trinucleotide |
| comp79133_c0_seq1 | NFKBIA | 324 | (A)^10 | 334 | Mononucleotide |
| comp79133_c0_seq1 | NFKBIA | 602 | (C)^10 | 612 | Mononucleotide |
| comp72310_c1_seq1 | P38 | 1 | (A)^15 | 16 | Mononucleotide |
| comp72310_c1_seq1 | P38 | 1521 | (G)^10 | 1531 | Mononucleotide |
| comp77558_c0_seq1 | PIK3C | 2303 | (GCT)^4 | 2315 | Trinucleotide |
| comp77940_c1_seq1 | PIK3C | 1717 | (T)^21 | 1738 | Mononucleotide |
| comp92187_c1_seq1 | PIK3C | 766 | (AAG)^4 | 778 | Trinucleotide |
| comp92187_c1_seq10 | PIK3C | 3312 | (AAG)^4 | 3324 | Trinucleotide |
| comp92187_c1_seq12 | PIK3C | 3195 | (AAG)^4 | 3207 | Trinucleotide |
| comp92187_c1_seq14 | PIK3C | 748 | (AAG)^4 | 760 | Trinucleotide |
| comp92187_c1_seq15 | PIK3C | 780 | (AAG)^4 | 792 | Trinucleotide |
| comp78505_c0_seq1 | PIK3R | 1 | (A)^20 | 21 | Mononucleotide |
| comp78505_c0_seq1 | PIK3R | 1102 | (A)^13 | 1115 | Mononucleotide |
| comp419325_c0_seq1 | PIK3R | 476 | (A)^10 | 486 | Mononucleotide |
| comp45723_c0_seq1 | TBK1 | 33 | (ATT)^4 | 45 | Trinucleotide |
| comp89291_c0_seq1 | TIRAP | 1053 | (AG)^6 | 1065 | Dinucleotide |
| comp95121_c0_seq1 | TLR1 | 2 | (AC)^11 | 24 | Dinucleotide |
| comp91305_c0_seq1 | TLR5 | 1431 | (GTT)^4 | 1443 | Trinucleotide |
