# Supplementary material for: Transcriptomic analysis of the host response to an iridovirus infection in Chinese giant salamander, Andrias davidianus
Source: Vet Res. 2015 Nov 20;46:136. doi: 10.1186/s13567-015-0279-8 (PMC4654921; doi:10.1186/s13567-015-0279-8)
Supplement: Supplementary file 8 — 10.1186/s13567-015-0279-8 SNPs analysis related to antiviral signaling pathway. This additional table provided a detail of SNPs involved in RIG-I-like receptor and the Toll-like receptor signaling pathways. The number of SNPs, which appeared in GSIV-infected (TS), uninfected (CS) and both of TS and CS samples, were also listed. [file 13567_2015_279_MOESM8_ESM.docx]

**Additional file 9 SNPs analysis related to antiviral signaling pathway**

|  | **CS** | **TS** | **same** | **Total** |  | **CS** | **TS** | **same** | **total** |
| --- | --- | --- | --- | --- | --- | --- | --- | --- | --- |
| RIG-I-like receptor signaling pathway | | | | | Toll-like receptor signaling pathway | | | | |
| ATG12 | 1 |  |  | 1 | AKT | 1 | 9 |  | 10 |
| TRAF6 | 1 |  |  | 1 | CCL3 | 1 |  |  | 1 |
| DDX3X |  | 2 |  | 2 | CD80 |  | 1 |  | 1 |
| MDA5 | 1 | 1 |  | 2 | CTSK | 2 | 1 |  | 3 |
| MITA | 1 | 2 |  | 3 | TIRP | 2 | 1 |  | 3 |
| PIN1 |  | 4 |  | 4 | CXCL9 |  | 4 |  | 4 |
| LGP2 | 1 | 7 | 1 | 9 | ERK1_2 |  | 1 | 1 | 2 |
| RIG-I | 7 | 2 | 2 | 11 | IL1B | 4 | 8 | 5 | 17 |
| CYLD | 5 | 7 |  | 12 | IRF5 | 6 | 1 |  | 7 |
| ISG15 | 2 | 1 | 13 | 16 | JUN |  | 1 |  | 1 |
| TRIM25 | 3 | 12 | 3 | 18 | MEK2 |  | 1 |  | 1 |
| OTUD5 |  | 52 |  | 52 | MAP3K7IP2 | 4 | 3 |  | 7 |
| **IRF3** |  | 1 |  | 1 | MYD88 | 1 |  |  | 1 |
| **NFKBIA** |  | 1 |  | 1 | PIK3C | 4 |  |  | 4 |
| **FADD** |  | 1 | 1 | 2 | PIK3R | 5 | 13 | 1 | 19 |
| **JNK** | 1 |  | 1 | 2 | RAC1 | 1 | 1 |  | 2 |
| **RELA** |  | 2 |  | 2 | SPP1 |  | 1 |  | 1 |
| **IL12B** |  | 3 |  | 3 | STAT1 | 3 |  |  | 3 |
| **CASP8** | 2 | 1 | 1 | 4 | TLR1 | 20 | 27 | 4 | 51 |
| **IKBKA** | 1 | 3 |  | 4 | TLR2 | 2 | 15 | 22 | 39 |
| **IRF7** | 1 | 1 | 2 | 4 | TLR3 |  | 1 |  | 1 |
| **IKBKE** | 2 | 1 | 4 | 7 | TLR6 | 32 | 18 | 27 | 77 |
| **P38** | 5 | 9 |  | 14 | TLR7 |  | 1 |  | 1 |
| **IL8** | 3 | 19 |  | 22 | TLR8 | 7 |  |  | 7 |
| **IKBKG** |  | 75 |  | 75 | TLR9 | 23 | 2 | 4 | 29 |
|  |  |  |  |  | CD40 | 5 | 1 |  | 6 |
|  |  |  |  |  | TOLLIP | 2 |  |  | 2 |
|  |  |  |  |  | TRAF6 | 1 |  |  | 1 |
|  |  |  |  |  | **IRF3** |  | 1 |  | 1 |
|  |  |  |  |  | **NFKBIA** |  | 1 |  | 1 |
|  |  |  |  |  | **FADD** |  | 1 | 1 | 2 |
|  |  |  |  |  | **JNK** | 1 |  | 1 | 2 |
|  |  |  |  |  | **RELA** |  | 2 |  | 2 |
|  |  |  |  |  | **IL12B** |  | 3 |  | 3 |
|  |  |  |  |  | **CASP8** | 2 | 1 | 1 | 4 |
|  |  |  |  |  | **IKBKA** | 1 | 3 |  | 7 |
|  |  |  |  |  | **IRF7** | 1 | 1 | 2 | 4 |
|  |  |  |  |  | **IKBKE** | 2 | 1 | 4 | 7 |
|  |  |  |  |  | **P38** | 5 | 9 |  | 14 |
|  |  |  |  |  | **IL8** | 3 | 19 |  | 22 |
|  |  |  |  |  | **IKBKG** |  | 75 |  | 75 |
| total | 37 | 207 | 28 | 272 | total | 141 | 228 | 73 | 445 |
