# Supplementary material for: Transcriptomic analysis of the host response to an iridovirus infection in Chinese giant salamander, Andrias davidianus
Source: Vet Res. 2015 Nov 20;46:136. doi: 10.1186/s13567-015-0279-8 (PMC4654921; doi:10.1186/s13567-015-0279-8)

**Additional file 6**

**A**: ESTs in the spleen library of Chinese giant salamanders hit to the RIG-I-like receptor signaling pathway.


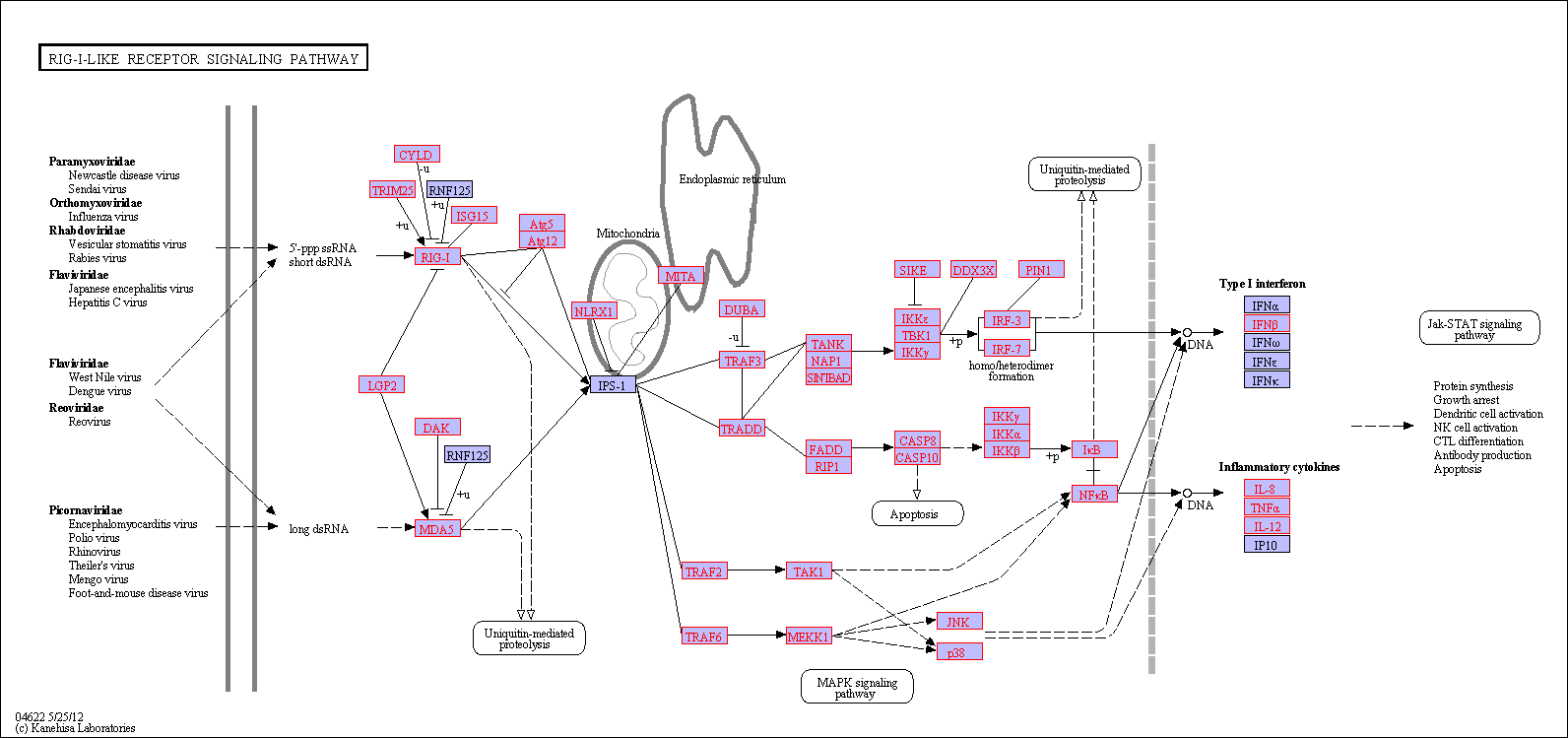


**B**: ESTs in the spleen library of Chinese giant salamanders hit to the complement and coagulation cascades.


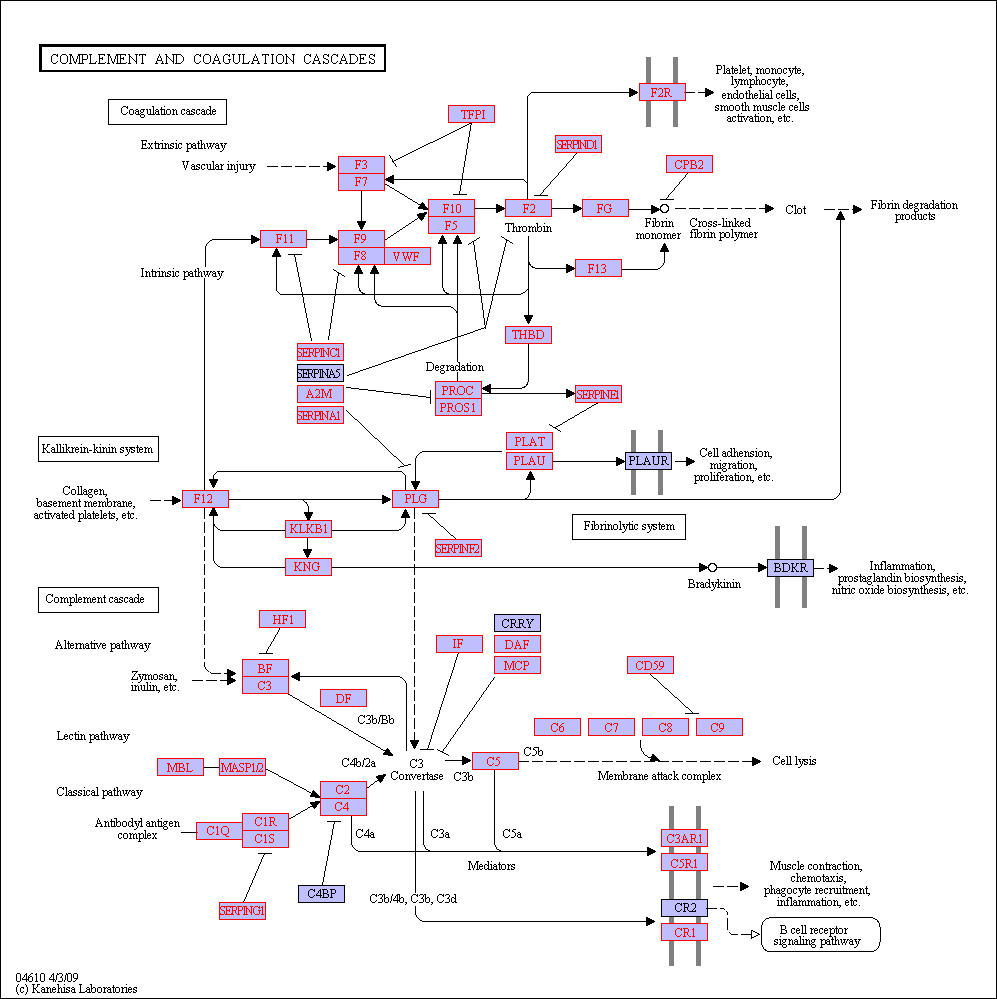

Supplement: Supplementary file 10 — 10.1186/s13567-015-0279-8 ESTs in the spleen library of Chinese giant salamanders hit to the RIG-I-like receptor signaling pathway (A) and complement and coagulation cascades (B). 44 ESTs including RIG-I, MDA5 and LGP2 were identified in the spleen library of Chinese giant salamanders, which hit to the RIG-I-like receptor signaling pathway (A). The majority of genes involved in the complement and coagulation cascades (53 of 59 total genes or 90%) were identified, and the alternative, lectin and classical pathways appeared conserved in the Chinese giant salamander (B). [file 13567_2015_279_MOESM10_ESM.docx]
