# Supplementary material for: Transcriptomic analysis of the host response to an iridovirus infection in Chinese giant salamander, Andrias davidianus
Source: Vet Res. 2015 Nov 20;46:136. doi: 10.1186/s13567-015-0279-8 (PMC4654921; doi:10.1186/s13567-015-0279-8)
Supplement: Supplementary file 11 — 10.1186/s13567-015-0279-8 Accession numbers of sequences used to build the phylogenetic tree. A total of 78 genes, which were used to build the phylogenetic tree, and their accession numbers were listed in this additional file. [file 13567_2015_279_MOESM11_ESM.docx]

**Additional file 11 Accession numbers of sequences used to build the phylogenetic tree**

| **Sequence name** | **Accession number** | **Sequence name** | **Accession number** |
| --- | --- | --- | --- |
| HsTLR1 | CAG38593 | MmNALP1 | AAR14736 |
| HsTLR2 | AAH33756 | HsNALP2 | AAG30289 |
| HsTLR3 | ABC86910 | MmNALP2 | NP_808358 |
| HsTLR4 | AAF05316 | HsNALP3 | AAL78632 |
| HsTLR5 | AAI09119 | MmNALP3 | AAR14737 |
| HsTLR6 | BAA78631 | HsNALP4 | NP_604393 |
| HsTLR7 | AAZ99026 | MmNALP4 | AAR14738 |
| HsTLR8 | AAZ95441 | HsNALP5 | NP_703148 |
| HsTLR9 | AAZ95520 | MmNALP5 | NP_035990 |
| HsTLR10 | AAY78491 | HsNALP6 | AAO18157 |
| MmTLR1 | AAI41322 | MmNALP6 | NP_598707 |
| MmTLR2 | AAF04277 | HsNALP7 | AAO18158 |
| MmTLR3 | AAH99937 | HsNALP8 | NP_789781 |
| MmTLR4 | NP_067272 | HsNALP9 | NP_789790 |
| MmTLR5 | NP_058624 | HsNALP10 | NP_789791 |
| MmTLR6 | BAA78632 | MmNALP10 | NP_780741 |
| MmTLR7 | AAI32386 | HsNALP11 | NP_659444 |
| MmTLR8 | NP_573475 | HsNALP12 | AAO18163 |
| MmTLR9 | AAK29625 | MmNALP12 | NP_001028603 |
| MsTLR11 | AAI09165 | HsNALP13 | NP_789780 |
| MmTLR12 | EDL30230 | HsNALP14 | NP_789792 |
| MmTLR13 | AAI17914 | MmNALP14 | NP_001002894 |
| DrTLR1 | AAI63271 | HsNOD1 | NP_006083 |
| DrTLR2 | AAQ90474 | DrNOD1 | XP_002665106 |
| DrTLR3 | AAI07956 | HsNOD2 | NP_071445 |
| DrTLR4a | ACE74929 | DrNOD2 | ADV31543 |
| DrTLR4b | AAQ90475 | HsNOD3 | DAA01245 |
| DrTLR5a | XP_001919052 | HsNLRC5 | NP_115582 |
| DrTLR5b | AAI63198 | MmNLRC5 | NP_001028379 |
| DrTLR6 | NP_001124065 | HsCIITA | CAA52354 |
| DrTLR7 | XP_003199309 | DrCIITA | XP_005163972 |
| DrTLR8b | XP_003199440 | HsAPAF1 | ABQ59028 |
| DrTLR9 | AAI63628 | DrAPAF1 | NP_571683 |
| DrTLR13L | XP_005167826 | HsNAIP | AAI43762 |
| DrTLR18 | AAI63840 | MmNAIP | AAD56762 |
| DrTLR19 | XP_002664892 | HsNLRC4 | AAH31555 |
| DrTLR21 | CAQ13807 | MmNLRC4 | NP_001028539 |
| DrTLR22 | AAI63527 | HsNLRX1 | AAI10891 |
| HsNALP1 | AAG30288 | DrNLRX1 | XP_005157652 |
